# Decomposition of outpatient health care spending by disease - a novel approach using insurance claims data

**DOI:** 10.1186/s12913-021-07262-x

**Published:** 2021-11-22

**Authors:** Michael Stucki, Janina Nemitz, Maria Trottmann, Simon Wieser

**Affiliations:** 1 ZHAW Zurich University of Applied Sciences, Winterthur Institute of Health Economics, Gertrudstrasse 15, 8401 Winterthur, Switzerland; 2 Department of Health Sciences and Medicine, University of Lucerne, Lucerne, Switzerland; 3 Helsana Insurance Group, Zürich, Switzerland; 4 SWICA Health Insurance, Winterthur, Switzerland

**Keywords:** Health care costs, Cost-of-illness, Outpatient care, Switzerland, Spending decomposition, I10

## Abstract

**Background:**

Decomposing health care spending by disease, type of care, age, and sex can lead to a better understanding of the drivers of health care spending. But the lack of diagnostic coding in outpatient care often precludes a decomposition by disease. Yet, health insurance claims data hold a variety of diagnostic clues that may be used to identify diseases.

**Methods:**

In this study, we decompose total outpatient care spending in Switzerland by age, sex, service type, and 42 exhaustive and mutually exclusive diseases according to the Global Burden of Disease classification. Using data of a large health insurance provider, we identify diseases based on diagnostic clues. These clues include type of medication, inpatient treatment, physician specialization, and disease specific outpatient treatments and examinations. We determine disease-specific spending by direct (clues-based) and indirect (regression-based) spending assignment.

**Results:**

Our results suggest a high precision of disease identification for many diseases. Overall, 81% of outpatient spending can be assigned to diseases, mostly based on indirect assignment using regression. Outpatient spending is highest for musculoskeletal disorders (19.2%), followed by mental and substance use disorders (12.0%), sense organ diseases (8.7%) and cardiovascular diseases (8.6%). Neoplasms account for 7.3% of outpatient spending.

**Conclusions:**

Our study shows the potential of health insurance claims data in identifying diseases when no diagnostic coding is available. These disease-specific spending estimates may inform Swiss health policies in cost containment and priority setting.

**Supplementary Information:**

The online version contains supplementary material available at 10.1186/s12913-021-07262-x.

## Introduction

Health care spending in rich countries like Switzerland is high and rising. Despite fierce debates on how to control health care spending, little is known about which diseases drive spending. Recent research has shown the potential of tracking disease-specific spending to explain changes in health care spending over time [1–10]. For Switzerland, the evidence is limited to a decomposition of total health care spending by 21 major diseases by Wieser et al. [11]. Their study also highlights the difficulties in identifying diseases in outpatient care, which is responsible for more than 50% of total health care spending [12].

The main objective of this paper is to decompose the spending for 12 outpatient services and drugs by 42 diseases or disease groups in 2017 in Switzerland. The second objective is to investigate the differences in disease-specific spending by age, sex, and type of health care service. The contributions are twofold: *First*, we use a multitude of diagnostic clues in insurance claims data to simultaneously identify a broad set of diseases. Previous research used Swiss administrative data and electronic health records to identify single diseases (e.g., multiple sclerosis [13], asthma/chronic obstructive pulmonary disease (COPD) [14] or diabetes mellitus [15]) or multiple diseases [16]. However, these studies covered only a limited number of diseases and used the type of drug as the only diagnostic clue. We also provide disease-specific spending estimates for Switzerland on a more granular level than in Wieser et al. [11]. *Second*, we apply a novel two-step decomposition method to assign individual spending to diseases. In the first step, we directly assign individual cost items to specific diseases if they are employed exclusively in the treatment of those diseases (e.g., anti-diabetic drugs for diabetes). In the second step, we use regression-based methods to distribute the remaining part of individual spending. Our comprehensive decomposition approach ensures that we allocate spending to one disease only. This is an advantage over single cost-of-illness studies that tend to over-estimate spending for the investigated disease [17].

By estimating spending by disease, age, sex, and service type, we provide the basis for a systematic and detailed health care spending monitoring.

### The Swiss health care system

The Swiss health care system offers timely access to a broad range of services. This comes at high costs, when compared to other high-income countries [18]. In 2018, total per capita health care spending according to National Health Accounts (NHA) was at 9420 Swiss Francs (CHF)^1^ [12], corresponding to 11.2% of GDP, the second highest share in the world after the United States [19]. Switzerland has universal coverage via mandatory health insurance (MHI). MHI covers a generous basket of health services but includes yearly deductibles and co-payments. Premiums are subsidized for low income households.

Outpatient care is highly fragmented and provided by a multitude of practitioners and hospitals on a fee-for-service basis. In contrast to inpatient care, there is no comprehensive diagnostic coding of outpatient care, e.g., by the international classification of diseases (ICD). Insurers are not allowed to collect information on the type of disease affecting their clients. However, they collect all bills for the services and drugs consumed by these clients. These claims data represent the most comprehensive source of information on outpatient service use in Switzerland. Nonetheless, claims data do not include all outpatient care spending: *First*, because MHI does not cover all outpatient services (e.g., most of dental care, except for unavoidable diseases of the chewing system or if treatment is associated with severe illness) and is not the only payer. The share of outpatient services covered by MHI was estimated at 67% of total outpatient spending [20]. *Second*, because bills for services covered by MHI might not be forwarded to health insurers, as individuals with high deductibles have to cover them anyway. Previous research has shown that unsubmitted claims amount to 2–3% of all claims [21].

### Diagnostic clues in tariff catalogues

The diagnostic clues that can be used to identify diseases consist of health services and drugs identified by specific tariff positions included in claims data. These positions are listed in five national tariff catalogues:

- The TarMed (tarif médical) for physician services contains about 4600 codes for either technical (e.g., thorax MRI) or time-dependent services (e.g., 5 min of consultation). Tariff points are assigned to each code and determine reimbursement together with the locally contracted price per tariff point.
- The AL (Analysenliste) for laboratory tests contains about 1800 codes and applies the same reimbursement mechanism as TarMed.
- The MiGel (Mittel- und Gegenständeliste) for therapeutic devices such as hearing aids contains about 700 codes and maximum prices for each device.
- The SL (Spezialitätenliste) for drugs contains about 9700 codes and the respective prices for all drug packages covered by MHI and classifies them according to the hierarchical anatomical therapeutic chemical (ATC) classification of the World Health Organization.
- The SwissDRG catalogue for inpatient acute care contains about 1000 codes based on the patient’s diagnoses and treatments.

## Data

We use data from SWICA, a major Swiss health insurer with a MHI market share of 8.1% in 2017 [22]. Our random sample of 709,788 insured covers 90% of the total SWICA insured population. A random sample of 90% of the insured population was taken to avoid showing results specifically for the enrolled population of SWICA in 2017. The sample is fairly representative of the general Swiss population with respect to the age/sex structure and the per capita MHI spending. The share of the elderly population is slightly lower than in the general population, and spending per capita is also slightly lower. Table 1 in Additional file 2 provides the descriptive statistics of this comparison.

**Table 1:** Number of clues used for disease identification by type of clue and disease level

	Level 2 diseases	Level 3 diseases
DRG codes (inpatient care)	333	290
ATC codes (drugs)	487	278
Physician specialization	18	2
Tariff catalogues		
TarMed	85	104
AL	0	9
MiGeL	22	19

In order to adjust for the slight differences in the age and sex structure, we computed weights for each 5-year age group and sex (21 × 2 = 42 groups) based on each group’s share in the sample and in the general population [23]. These weights were used in the estimation of the overall prevalence rates as well as the computation of the spending by disease, age group, sex, and service type.

The sample consists mainly of individuals insured with SWICA for the entire year 2017, as individuals may switch to another MHI provider only at the beginning of each year. We observe total spending in MHI at the individual level by service provider (e.g., general practitioner) and service (e.g., physician services). The spending includes the part borne by the insurer as well as deductibles and the co-payments covered by insurees, provided that the bill was sent to the health insurer. The analysis was performed from a health insurance perspective, including co-payments and deductibles paid by insurees.

In addition to the spending by service, the data contains the number and the billed amount of selected tariff positions from the tariff catalogues. These tariff positions were used in the disease identification.

## Method

We proceeded in three steps. *First*, we defined a decomposition framework consisting of a comprehensive health service and disease classification. *Second*, we developed a disease identification algorithm and used it to label individuals with specific diseases. This allowed us to estimate the treated prevalence of each disease. *Third*, we assigned the spending at the individual level to diseases, either directly or regression-based. This allowed us to estimate the total outpatient treatment costs of each disease. The following section describes these steps in more detail.

### Decomposition framework

We defined a comprehensive and mutually exclusive set of 12 outpatient services using the classification of the Swiss NHA from 2017 [12]. The 12 outpatient services include general practitioners (GPs), specialist physicians, outpatient hospital, drugs, home care, physiotherapy, occupational therapy, outpatient psychiatry, laboratory tests performed by external laboratories outside the doctor’s office, radiology, dental care (for the few indications covered by MHI), and other spending (e.g., devices). Spending by disease was estimated separately for each service.

We classified diseases according to the Global Burden of Disease (GBD) study [24]. This has four major advantages: *First*, the classification is mutually exclusive, thus avoiding double counting. *Second*, the GBD provides prevalence rates, which can be used to validate our results. *Third*, the GBD estimates mortality and disability-adjusted life years (DALY) by disease, which may be used to complement spending by disease with the disease burden. *Fourth*, it enables comparisons with other studies using the same classification [11, 25].

The GBD classification comprises four hierarchical disease levels with 359 diseases and injuries at the most granular level. Level 1 distinguishes between injuries, non-communicable diseases (NCDs), and ‘other diseases’ including communicable diseases. Level 2 distinguishes between major diseases groups (such as ‘cardiovascular diseases’) and levels 3 and 4 distinguish by more specific diseases (e.g., ‘stroke’ and ‘ischemic stroke’).

We used a simplified GBD classification, as many diseases could not be identified due to a lack of specific diagnostic clues in claims data.

Whenever possible, we defined diseases at GBD level 3. For communicable diseases, we only distinguished between GBD level 2 diseases, except for two GBD level 3 diseases (HIV/AIDS and hepatitis). For NCDs, we selected between two and four GBD level 3 diseases for each major disease group at GBD level 2. The selection of diseases was based on two principles: *First*, the prevalence level of the disease according to the GBD study for Switzerland in 2017 [26]. For neoplasms, the four localizations were chosen based on incidence rates as reported by the national cancer registry NICER [27]. *Second*, the availability of clues in the claims data to identify diseases. We judged this availability based on knowledge from previous studies. After selecting diseases within each category, we summed up the others in the residual category (e.g., ‘other cardiovascular diseases’).

Ultimately, our exhaustive und mutually exclusive classification consisted of 42 diseases at GBD level 3 and 15 major disease categories at GBD level 2. As in previous research [11], we added a ‘well care’ category for health care spending on healthy pregnancies, preventive check-ups and other non-diseases. We excluded injuries, as treatment of injuries is financed by a separate mandatory accident insurance.

### Disease identification in claims data

We developed a disease identification algorithm to identify diseases based on the diagnostic clues contained in claims data. The algorithm was then used to label individuals with specific diseases. This procedure was based on clinical literature, information on the medical indication of procedures and drugs, and advice from clinical experts. The algorithms consisted of single clues (e.g., specific billing positions), or a combination of clues (e.g., a specific billing position and physician specialization). When clues allowed a disease identification only at GBD level 2 but not at level 3, individuals were assigned to the residual ‘other’ disease category at level 3.

The DRG codes from inpatient care served as reliable disease clues when they directly linked to specific ICD-10 codes. We explored the degree of correspondence between the DRG codes and ICD-10 codes in the Swiss inpatient registry [28], which covers all inpatient care episodes. We set a minimum of a 95% correspondence between DRG and ICD-10 codes to include a DRG code in the disease algorithm. The DRG code B69D (Transient ischemic attack and extracranial vascular occlusions) did for example correspond to a main diagnosis of a stroke (ICD-10: I60-I69) in 99.8% of cases and was therefore used for identification.

As in previous studies [16, 29], ATC drug codes allowed for the identification of numerous diseases. This was very effective when the treatment called for a disease-specific drug (e.g., drugs for HIV).

Physician specialization (e.g., oncology) was mostly used to identify GBD level 2 diseases but was particularly useful when linked to exclusive disease groups, such as oncologists for cancer.

Single tariff codes from the AL and TarMed included the repeated use of prostate-specific antigen (PSA) testing to identify prostate carcinoma and TarMed chapter 23.02 (tumour surgery of the mamma) to identify mamma carcinoma (both in combination with more clues referring to cancer).

Table 1 summarizes the number of clues used for disease identification by type of clue and disease level. The algorithms used to identify diseases are provided in the Additional file 1.

### Spending assignment

The last step after labelling individuals with specific diseases was to assign spending at the individual level to diseases. This is somewhat challenging, as individuals may suffer from multiple diseases and spending due to each disease must be estimated appropriately [30]. Several encounter-, episode- and person−/regression-based methods have been proposed for this spending allocation [17].

We implemented a novel two-step spending assignment procedure. The main innovation is the direct assignment of detailed spending items to diseases, followed by an indirect (regression-based) assignment of the residual individual spending.

Spending for each individual *i* by service *s*, ${y}_{i,s}^{total}$, was decomposed and assigned to diseases in two steps: *First*, we directly assigned spending to disease *d* using disease clues that uniquely identified diseases at GBD level 3 (e.g., ATC codes), yielding ${y}_{i,s,d}^{direct}.$ In other words, whenever a single claim (e.g., a specific drug) indicated a specific disease (e.g., prostate cancer), its spending was directly allocated to that disease. Spending associated with specific clues used at disease level 2 (e.g., physician specialization) was distributed equally to the level 3 disease(s) within the corresponding level 2 group. *Second*, we used regression-based attributable fractions (AF) to decompose the residual spending ${y}_{i,s}^{residual}={y}_{i,s}^{total}-\sum \limits_{j=1}^{42}{y}_{i,s,j}^{direct}$ [31], thereby following previous studies [32–34]. This allowed for the assignment of non-disease-specific claims (e.g., outpatient spending in hospitals). We ran regressions of spending on all 42 disease indicators, separately for 56 groups that were defined based on outpatient service category (7 services^2^), sex (male/female), and age (4 groups: < 20 y./20–44 y./45–64 y./65+ y.).

We estimated a Poisson pseudo-maximum likelihood (PPML) model, which has been shown to perform especially well when there are many zeros in the dependent variable [35]^3^:

$$ \Pr \left({y}_{i,s}^{residual}|{I}_{i,1},\dots, {I}_{i,42}\right)=\frac{e^{-{\lambda}_{i,s}}\left({\lambda}_{i,s}^{y_{i,s}^{residual}}\right)}{y_{i,s}^{residual}!} $$

where

$$ {\lambda}_{i,s}={e}^{\alpha_s+\sum \limits_{j=1}^{42}{\beta}_{s,j}{I}_{i,j}+{\varepsilon}_{i,s}} $$

and *I*_*i*, *j*_ is an indicator equal to 1 if disease *j* is present in insured *i* and 0 if not.

The AF is the part explained by the disease indicators. The exponentiated constant *α* in our models represents the estimated mean spending with all disease indicators equal to 0. The spending share *s*_*i*, *s*, *d*_ for each disease *d* for insured *i* was calculated using the regression coefficients *β*_*s*, *j*_. Multiplying the AF with *s*_*i*, *s*, *d*_ and the residual spending resulted in the spending for disease *d*.

$$ A{F}_{i,s}=\frac{\hat{y_{i,s}}-{e}^{\upalpha_s}}{\hat{y_{i,s}}} $$

$$ {s}_{i,s,d}=\frac{\left({e}^{\upbeta_{s,d}}-1\right)\ast {I}_{i,d}}{\sum_{j=1}^{42}\left[\left({e}^{\upbeta_{s,j}}-1\right)\ast {I}_{i,j}\right]} $$

$$ spendin{g}_{i,s,d}=A{F}_{i,s}\ast {s}_{i,s,d}\ast {y}_{i,s}^{residual} $$

We only assigned spending to a disease if its coefficient was significant at the 5% level. Furthermore, we did not allow for any negative effects on individual spending, i.e. we set negative *β* to zero.^4^

The share of observations with zero spending was very high for home care. We therefore did not apply the regression-based approach for home care, but split spending at the individual level equally across all the identified diseases. We applied a similar logic to the spending for occupational therapy and split it across all identified neurological and musculoskeletal disorders.

### Validation and robustness checks

The validation of our disease identification approach is challenging, due to the lack of diagnostic coding in outpatient care. Nonetheless, we compared the disease prevalence rates in our sample with the existing literature. Furthermore, we performed an internal validation by running the disease identification and spending assignment procedure on ten random subsamples each consisting of 70% of the full sample.

The spending by disease depends substantially on the number of patients with the disease. We thus checked the robustness of the spending decomposition results by running a set of scenarios with different thresholds for disease identification based on two clues: drug consumption (i.e., number of packages per year) and treatment by a physician with a certain specialization (i.e., the total spending for a physician with a certain specialization). We defined nine scenarios, combining three different thresholds: at least 1, 2, or 3 drugs with ATC codes used for the disease, as well as at least 1, 300, or 1000 CHF spending for a service provider (e.g., oncologist for the identification of neoplasms) over the course of the year. The scenario with the two lowest thresholds (min. 1 package, min. 1 CHF with service provider) was the one used for most diseases in the disease identification and spending assignment procedure described in 3.2 and 3.3. Only for some diseases (e.g., chronic respiratory diseases) we defined higher thresholds (for additional information on the thresholds used see Additional file 1).

## Results

### Treated prevalence of identified diseases

We identified 14 major disease groups on GBD level 2 and 42 more specific diseases at GBD level 3, with the addition of well care at both levels of the classification (see 1st and 2nd column in Table 2). The treated prevalence rates were calculated using age group and sex specific weights of the Swiss population (see 3rd column in Table 2).

**Table 2:** Estimated treated prevalence rates of identified diseases and comparison with other estimates

GBD level 2	GBD level 3	Prevalence estimate	GBD prevalence estimate [26]	Other source
**Communicable diseases (total)**		19.98%	24.27%	
	HIV, AIDS	0.17%	0.27%	
	Hepatitis	0.03%	0.09%	
	Other communicable	19.73%	n.a.	
**Maternal and neonatal disorders**	Maternal and neonatal disorders	0.21%	0.94%	
**Nutritional deficiencies**	Nutritional deficiencies	5.37%	4.17%	
**Neoplasms (total)**		1.83%	12.53%	1.48% [36]
	Colon and rectum cancer	0.27%	0.42%	0.17% [36]
	Trachea, bronchus and lung cancer	0.14%	0.08%	0.10% [36]
	Breast cancer	0.40%	0.85%	0.32% [36]
	Prostate cancer	0.24%	0.74%	0.30% [36]
	Other cancers	1.07%	n.a.	n.a.
**Cardiovascular diseases (total)**		16.92%	9.86%	
	Ischemic heart disease	2.14%	3.20%	
	Stroke	0.31%	1.11%	
	Hypertensive heart disease	0.08%	0.19%	
	Atrial fibrillation and flutter	0.46%	1.45%	0.94% (canton of Geneva) [37]
	Other cardiovascular	14.13%	n.a.	
**Chronic respiratory diseases (total)**		4.54%	11.54%	
	Chronic obstructive pulmonary disease (COPD)	0.80%	5.06%	2.60–4.50% (age > 30) [14]
	Asthma	3.32%	7.23%	5.10–6.40% (age > 30) [14]
	Other chronic respiratory	0.90%	n.a.	
**Digestive diseases (total)**		10.27%	21.52%	
	Cirrhosis and chronic liver disease	0.03%	13.67%	
	Other digestive	10.24%	n.a.	
**Neurological disorders (total)**		10.99%	43.27%	
	Alzheimer’s and dementia	1.37%	1.60%	4.50% (age > 59) [38]
	Parkinson’s	0.67%	0.27%	0.91% (age > 59) [38]
	Epilepsy	1.93%	0.37%	
	Multiple Sclerosis	0.07%	0.13%	
	Other neurological	7.12%	n.a.	
**Mental and substance use disorders (total)**		11.86%	21.40%	26.00–28.30% (age 18–65) [39]
	Schizophrenia	0.20%	0.37%	0.39% [40]
	Depression	5.10%	4.74%	4.40% [41]
	Attention Deficit Hyperactivity Disorder (ADHD)	0.62%	1.18%	
	Alcohol and drug use disorders	0.24%	4.04%	3.30% (only alcohol) [42]
	Other mental	6.10%	n.a.	
**Diabetes and kidney disease (total)**		4.41%	16.35%	
	Diabetes mellitus	3.63%	8.20%	6.30% [43]
	Chronic kidney disease	0.14%	11.36%	10.40% [44]
**Skin and subcutaneous diseases**	Skin and subcutaneous diseases	16.05%	34.45%	
**Sense organ diseases**	Sense organ diseases	24.22%	20.57%	
**Musculoskeletal disorders (total)**		27.57%	34.67%	
	Rheumatoid arthritis	0.63%	0.48%	
	Osteoarthritis	2.50%	13.15%	
	Low back pain	0.85%	18.63%	24.30% [45]
	Osteoporosis	3.12%	n.a.	13.80% (age > 39) [46]
	Other musculoskeletal	21.43%	n.a.	
**Other non-communicable diseases (total)**		14.11%	75.62%	
	Oral disorders	2.37%	55.23%	
	Other non-communicable	12.20%	n.a.	
**Well care**	Well care	20.94%	n.a.	

Note: The prevalence estimates based on the study sample were weighted by age/sex-specific weights

**Fig. 1:**
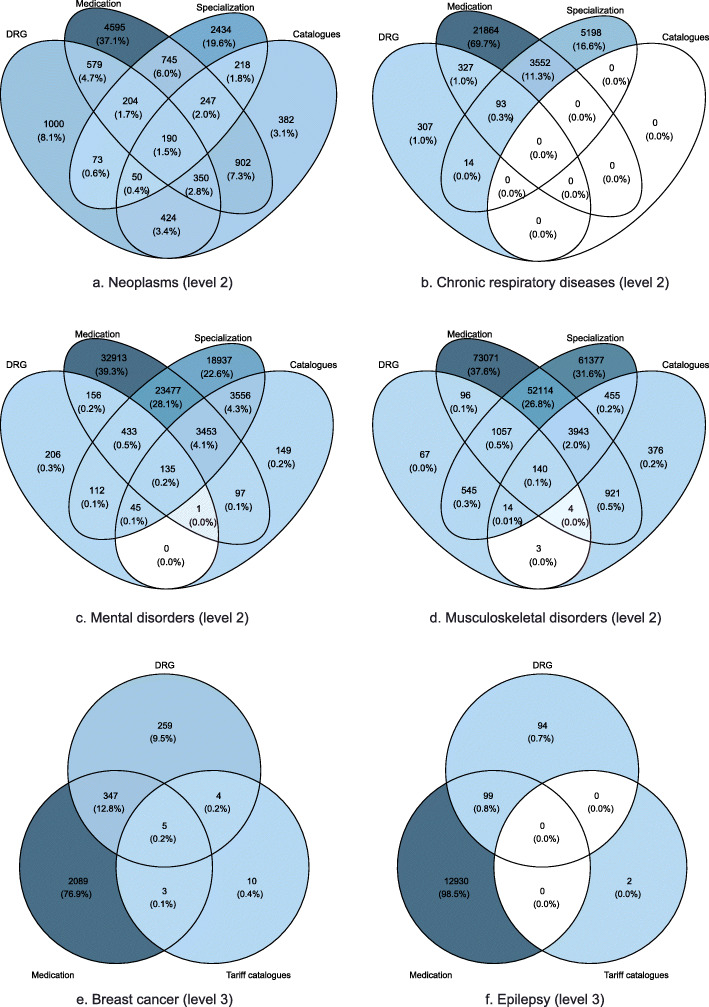
Venn diagrams of clues used in the identification of diseases. Numbers refer to the number of patients identified with the clues (in parentheses: share within each disease)

Disease clues overlapped substantially in disease identification, as individuals identified as prevalent cases of a disease based on one clue were often also identified by other clues. Diseases at level 2 were identified based on one (80.1% of prevalent patients), two (17.5%), three (2.3%), or all four (0.1%) types of clues. For level 3, the corresponding shares were 87.9, 10.7, 1.3, and 0.006%. Diseases were sometimes identified by several clues of the same type (e.g., different drugs). The degree of the overlap between types of clues is illustrated by the Venn diagrams in Fig. 1 for selected diseases. The figure shows that both type and number of disease clues vary across diseases. Table 2 in the Additional file 2 in the Appendix shows the share of patients by the number of clues for each disease.

### Spending by disease

**Fig. 2:**
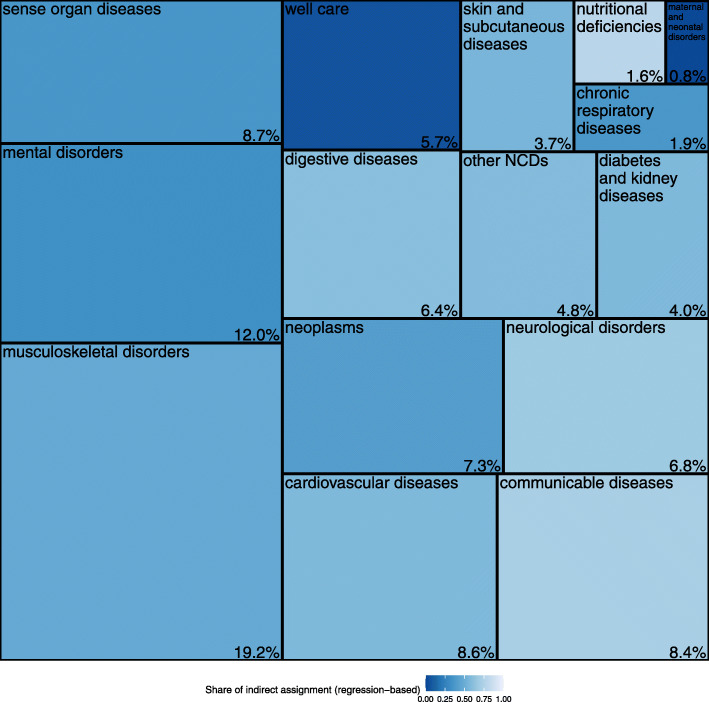
Outpatient spending by disease at GBD level 2 (% of total spending assigned)

We were able to assign 80.7% of outpatient spending to diseases. Of these, 53.5% were directly assigned (first step) and 46.5% indirectly assigned (regression-based second step). The regression coefficients used in the computation of the AF are shown in Additional File 3. 19.3% of outpatient spending could not be allocated to any disease. This is for two reasons: first, spending of people who were not flagged with any disease (3.7%) and second, the share of residual spending not assigned in the regression procedure (15.6%). Figure 2 shows the shares of assigned outpatient spending by level 2 diseases. The largest share was devoted to musculoskeletal disorders (19.2%), followed by mental disorders (12.0%), sense organ diseases (8.7%) and cardiovascular diseases (8.6%). The brighter the area, the more was assigned indirectly in the second step.

**Fig. 3:**
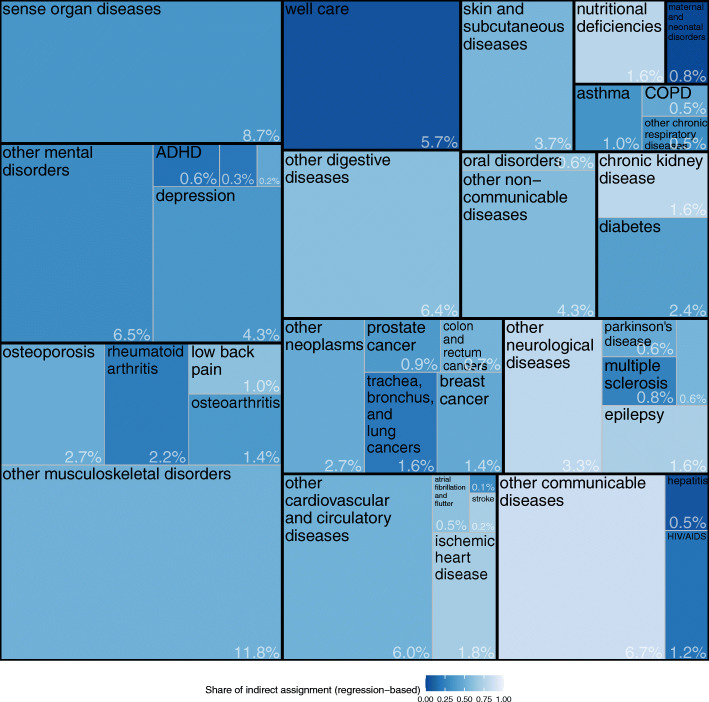
Outpatient spending by disease at GBD level 3 (% of total spending assigned). Note: Labelling of selected diseases with low spending shares was omitted for reasons of readability

Figure 3 shows the results by level 3 diseases. Depression had the largest share within mental diseases, with 4.3% of total outpatient spending. The most expensive type of cancer was trachea, bronchus, and lung cancer (1.6%). Other costly single diseases were osteoporosis (2.7%), diabetes (2.4%), and rheumatoid arthritis (2.2%).

### Differences in spending by age, sex and service

#### Spending by disease and sex

**Fig. 4:**
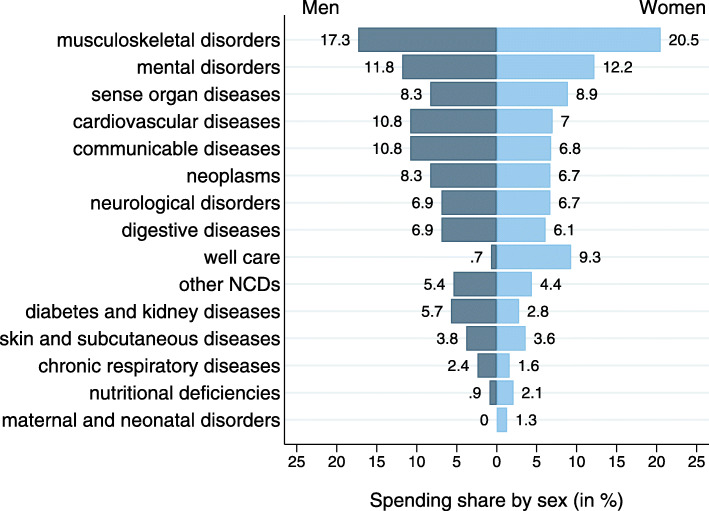
Outpatient spending by disease groups for men and women separately (% of total assigned spending). Note: Spending shares refer to the spending that could be assigned. NCDs: non-communicable diseases

Spending shares by disease were similar for both men and women, with few exceptions (Fig. 4). Well care (0.7% for men/9.3% for women) was more relevant in women, mainly due to spending for healthy pregnancy. Men showed higher spending for cardiovascular (10.8%/7.0%), chronic respiratory (2.4%/1.6%), and communicable diseases (10.8%/6.8%) as well as diabetes and kidney diseases (5.7%/2.8%). On the other hand, women showed higher relative spending for musculoskeletal (17.3%/20.5%) and nutritional diseases (0.9%/2.1%). The results for level 3 diseases are shown in Fig. 1 in the Additional file 2.

#### Spending by disease and age

**Fig. 5:**
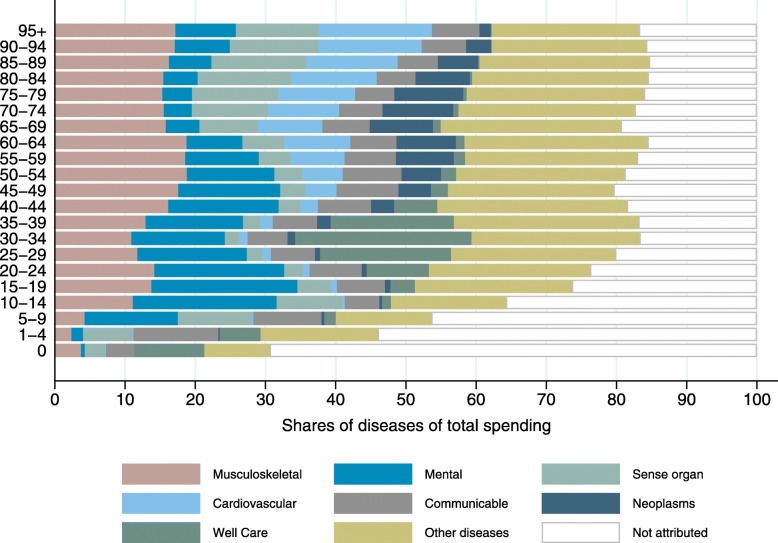
Outpatient spending by major disease groups (in % of total by age group)

Figure 5 shows spending on major disease groups by five-year age groups. Younger age groups had a substantially higher spending share for mental diseases. Cardiovascular diseases had a higher spending share in older age groups. The same was true for neoplasms, which show, however, a decreasing spending share in patients above age 80. The share of spending that was not attributed to any disease was much higher in young individuals. Absolute spending by disease and age groups is shown in Fig. 2 in the Additional file 2.

**Fig. 6:**
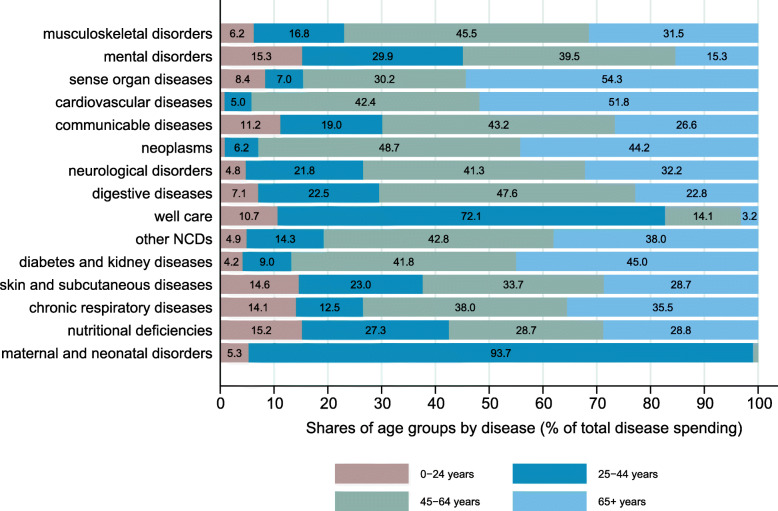
Outpatient spending of broad age groups by disease group (% of assigned spending)

The spending share attributable to age groups varied by disease (Fig. 6). The population above age 65 consumed more than half of the spending assigned to sense organ and cardiovascular diseases. In other disease groups like musculoskeletal, mental, and neurological disorders, the predominant group were individuals aged 45–64 years.

#### Spending by disease and health care service

**Fig. 7:**
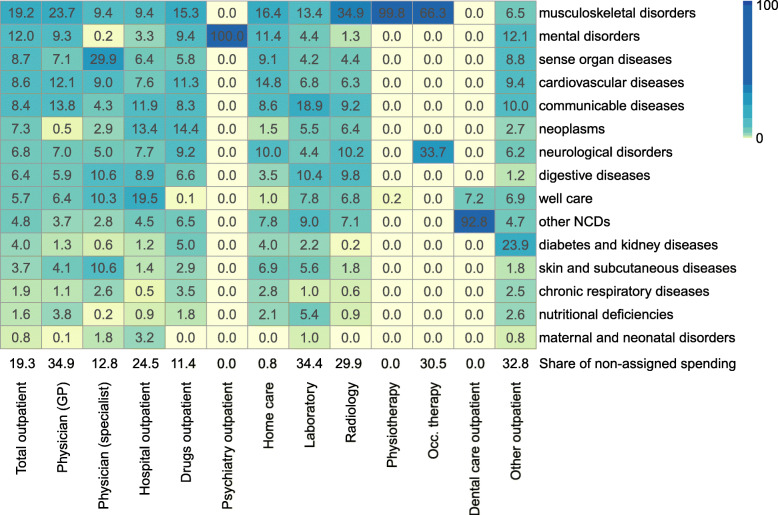
Outpatient spending by disease groups at GBD level 2 and health service (% of spending assigned to health services)

The spending by disease differed significantly across services. As illustrated in Fig. 7 the spending share of neoplasms was higher in hospital outpatient care (13.4%) and drugs (14.4%). Musculoskeletal disorders showed the highest spending shares in many services, such as GPs (23.7%), drugs (15.3%), home care (16.4%), and radiology (34.9%). The numbers in the coloured area of the figure refer to the assigned spending and add up to 100%. The part that could not directly be allocated in the two assignment steps is shown below the coloured area above the health service labels. Non-assigned spending was highest for GPs (34.9%) and equal to zero for services that we assigned completely to diseases (e.g., psychiatry). The share was also low for specialists (12.8%) and drugs (11.4%).

**Fig. 8:**
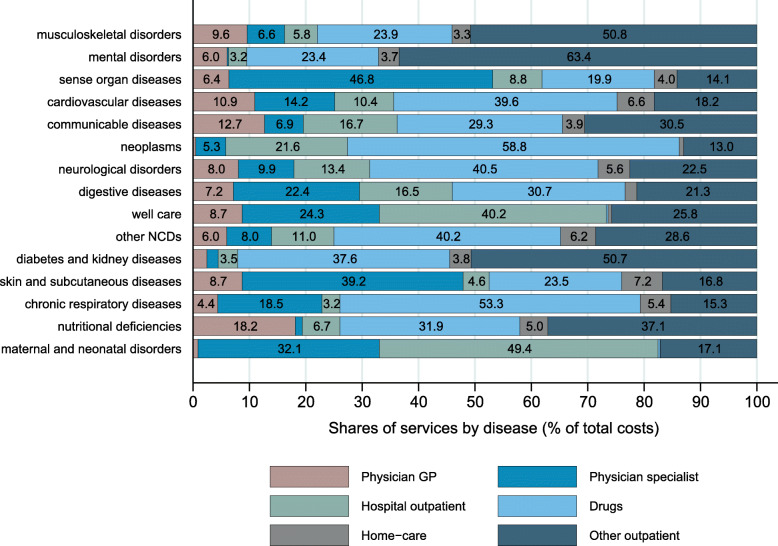
Spending shares of outpatient services by disease group at GBD level 2 (% of spending assigned to diseases). GP: General practitioner. Note: the category “other outpatient” comprises all the outpatient services not explicitly shown in the graph (physiotherapy, occupational therapy, psychiatry, radiology, laboratory tests, dental care, other outpatient)

The relative importance of services in the estimated spending varied by disease (Fig. 8). Drugs were the most important spending component for many diseases (e.g., neoplasms and chronic respiratory diseases). The high shares of other outpatient care in mental disorders and diabetes and kidney disease was mainly due to psychotherapy (mental) and dialysis (kidney disease). The results for spending by services and diseases at level 3 are shown in Figs. 3 and 4 in the Additional file 2.

### Average annual disease-specific spending per patient

Average disease-specific spending per patient was calculated by dividing total spending by disease by the number of patients with that disease. This disease-specific spending is an average over all prevalent patients treated for that disease in 2017. It thus represents an average over incident and prevalent cases of different disease stages. Table 3 shows this spending per patient separately for women and men (column 3 and 4). Average annual spending per patient was highest for hepatitis, followed by trachea, bronchus and lung cancer, and chronic kidney disease. Columns 5 and 6 in the table show the overall share of women and men in disease specific spending. The last column shows the share of total outpatient spending for that disease.

**Table 3:** Outpatient spending by disease at GBD level 3 (share by sex, per patient by sex, overall share)

Disease GBD level 2	Disease GBD level 3	Average annual disease specific spending per patient (CHF)		Spending share by sex (%)		Share in total spending (%)
		women	men	women	men	
**Communicable diseases**	HIV/AIDS	14,296	14,908	24.6	75.4	1.2
	Other communicable diseases	610	879	51.7	48.3	6.7
	Hepatitis	38,145	41,050	42.4	57.6	0.5
**Maternal and neonatal disorders**	Maternal and neonatal disorders	7982	562	99.8	0.2	0.8
**Nutritional deficiencies**	Nutritional deficiencies	572	1130	76.3	23.7	1.7
**Neoplasms**	Colon and rectum cancers	3977	8988	44.8	55.2	0.7
	Trachea, bronchus, and lung cancers	22,969	26,083	54.2	45.8	1.6
	Breast cancer	7645	0	100.0	0.0	1.4
	Prostate cancer	0	7743	0	100.0	0.9
	Other neoplasms	5151	6552	47.3	52.7	2.7
**Cardiovascular diseases**	Ischemic heart disease	1452	2040	41.3	58.7	1.8
	Stroke	1752	1499	50.3	49.7	0.2
	Hypertensive heart disease	3843	2155	58.5	41.5	0.1
	Atrial fibrillation and flutter	2058	2171	42.0	58.0	0.5
	Other cardiovascular and circulatory diseases	849	943	50.1	49.9	6.0
**Chronic respiratory diseases**	Chronic obstructive pulmonary disease (COPD)	1102	1332	44.9	55.1	0.5
	Asthma	609	640	51.1	48.9	1.0
	Other chronic respiratory diseases	1267	1121	45.1	54.9	0.5
**Digestive diseases**	Cirrhosis and other chronic liver diseases	1494	3428	23.0	77.0	0.0
	Other digestive diseases	1341	1283	55.5	44.5	6.4
**Neurological disorders**	Alzheimer’s disease and other dementias	926	947	62.3	37.7	0.6
	Parkinson’s disease	1653	2280	49.6	50.4	0.6
	Epilepsy	1614	1828	52.8	47.2	1.6
	Multiple sclerosis	22,429	20,381	71.6	28.4	0.8
	Other neurological diseases	947	981	58.0	42.0	3.3
**Mental and substance use disorders**	Schizophrenia	3184	4041	43.5	56.5	0.3
	Depression	1746	1868	63.7	36.3	4.3
	Attention Deficit Hyperactivity Disorder (ADHD)	2419	1911	41.4	58.6	0.6
	Other mental disorders	2298	2197	60.0	40.0	6.5
	Alcohol and drug use disorders	1963	2107	38.4	61.6	0.2
**Diabetes and kidney diseases**	Diabetes	1398	1417	43.0	57.0	2.4
	Chronic kidney disease	22,754	25,603	38.7	61.3	1.6
**Skin and subcutaneous diseases**	Skin and subcutaneous diseases	473	498	56.8	43.2	3.7
**Sense organ diseases**	Sense organ diseases	790	707	60.4	39.6	8.7
**Musculoskeletal disorders**	Rheumatoid arthritis	7148	7721	57.0	43.0	2.2
	Osteoarthritis	1247	1113	67.9	32.1	1.4
	Low back pain	2666	2335	63.3	36.7	1.0
	Osteoporosis	1741	2350	79.0	21.0	2.7
	Other musculoskeletal disorders	1234	1072	59.3	40.7	11.8
**Other non-communicable diseases**	Oral disorders	478	511	57.1	42.9	0.6
	Other non-communicable diseases	715	779	53.0	47.0	4.3
**Well care**	Well care	676	148	95.1	4.9	5.7
**Not assigned**		606	632	53.7	46.3	–

*The table shows the main results of the spending decomposition by disease: the average annual spending per patient with the disease for women (column 3) and men (column 4) as well as the share of total outpatient spending by disease by women and men (columns 5 and 6). The last column refers to the share of total outpatient spending that was assigned to that disease. The last row represents the average spending per individual with positive spending that could not be assigned*

### Validation and robustness of results

In Table 2, we provide values from the GBD study for Switzerland (4th column) [26] and from other previous epidemiological literature for Switzerland (5th column). The best available data source to compare our prevalence estimates with is registry data, which is available only for neoplasms in the national cancer registry NICER [36].

The internal validation of the spending estimates by diseases showed that the spending shares of diseases were very similar across subsamples. Running the disease identification and the subsequent spending assignment on ten randomly drawn subsets showed that the spending shares ranged between +/− 0.3%-points at the maximum (+/− 0.1%-points on average) compared to the initial estimates for diseases at level 2.

**Fig. 9:**
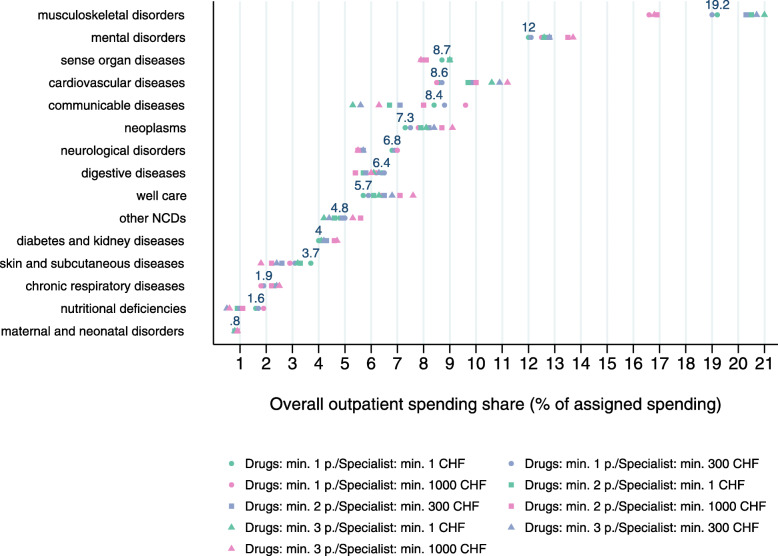
Spending shares in scenario analysis for GBD level 2 diseases. Note: The scenarios include different combinations of minimum requirements of drug utilization (number of packages) and spending at a physician with a certain specialization used in the identification of diseases. Spending shares refer to the spending that could be assigned. The numbers shown for each disease refer to spending shares in the base estimation. NCDs: non-communicable diseases

The nine scenarios that we ran to check for the robustness of our results led to different spending shares by disease group (see Fig. 9). For most diseases, the shares were similar across the scenarios. The largest variation was observed for communicable diseases (min: 5.3, max: 9.6), while the shares for mental disorders varied only slightly (min: 12.0, max: 13.7). The three scenarios with the highest specialist spending threshold (in pink) decreased the share of musculoskeletal disorders quite substantially. The minimum, maximum, and mean values for all level 2 and level 3 diseases are shown in Tables 4 and 5 in Additional file 2.

## Discussion

This study estimated disease-specific outpatient spending by age, sex, and health care services and drugs in Switzerland in 2017. Diseases were classified based on a simplified exhaustive and mutually exclusive GBD classification, with two hierarchical levels including 15 major disease groups at GBD level 2, and 42 more specific diseases at GBD level 3. Health care services and drugs were classified based on a simplified National Health Accounts classification. Diseases were identified based on a combination of diagnostic clues in claims data from a major MHI provider. Spending was assigned to diseases both directly and indirectly (regression-based).

### Interpretation of results

Our results show the number of individuals treated in outpatient settings for specific diseases in 2017, as well as the disease-specific outpatient spending (overall and per patient) in that year. Our estimated treated prevalence rates are mostly lower than the overall prevalence rates estimated in the GBD study for Switzerland and in other studies (see Table 2). This difference may be explained by two factors: *First*, not all individuals affected by a disease in a year (prevalent cases) are treated for the disease in that year. Possible reasons include lack of diagnosis, lack of access to care, lack of treatment options, and lack of current need of treatment, as well as the patient’s choice not to be treated. *Second*, even if the individual was treated for a disease, we could not identify the disease if we had no suitable diagnostic clue in the claims data. The first factor does not affect our spending by disease estimations, as health care spending can only occur when patients are treated. The second factor will, however, lead to an underestimation of disease-specific spending.

In general, our disease identification algorithms performed reasonably well in diseases with specific treatments and compelling need of treatment, such as diabetes, ischemic heart disease or lung cancer. The identification was more challenging for diseases with mainly unspecific treatments, such as low-back pain and osteoarthritis. However, these diseases could in part be identified at the GBD level 2 of the major disease categories, such as musculoskeletal disorders and mental and substance use disorders.

### Reasons for differences in prevalence rates

The differences between our estimates and GBD prevalence rates may be explained. *First*, we identified treated prevalence, while GBD reports overall prevalence. *Second*, the GBD prevalence estimates are not always derived from epidemiological data from Switzerland, but from other countries. *Third*, the identification for some diseases was difficult with our data, because treatments were usually not covered by MHI, e.g., oral disorders.

### Spending by disease

We were able to assign 80.7% of outpatient spending to diseases. We found that almost half of outpatient spending was on just four disease groups: musculoskeletal disorders (19.2% of total assigned spending), mental and substance use disorders (12.0%), sense organ diseases (8.7%), and cardiovascular diseases (8.6%). These often chronic diseases are highly prevalent, in many cases well treatable and do usually not lead to death (with the exception of cardiovascular diseases). Neoplasms accounted for 7.3% of outpatient spending, although they had the largest health burden in terms of years of life lost in 2017 (46.6% of total in women and 28.5% in men) [47]. This may be explained by the often short and acute rather than chronic disease episodes, leading to lower overall spending, even if spending per patient may be very high. Previous literature has found similar results for neoplasms [11, 25].

The share of direct spending assignment differed substantially across diseases. It was high when we had either specific drugs with a large share in disease-specific spending (e.g., for communicable diseases, multiple sclerosis, or ADHD) or when we were able to directly identify all reimbursed services (e.g., healthy pregnancy or prevention services for well care). The share of non-assigned spending was quite low for drugs (11.4%), as spending for many expensive drugs could be assigned directly to specific diseases.

### Spending by sex, age, and type of health care service

We observed substantial differences in the disease-specific spending by service type. The relative importance of single diseases for each type of health service is strongly influenced by the type of available outpatient treatment options. The highest share of spending on drugs is for example for neoplasms and musculoskeletal disorders, while the highest share of spending on laboratory tests is for communicable diseases.

There were substantial differences in spending by disease across age groups and sex. Spending on mental and substance use disorders was considerably higher in the younger age groups, while spending on cardiovascular and sense organ diseases was considerably higher in the older age groups. A higher share of spending could not be assigned to any disease in the younger age groups. This may indicate that younger individuals are relatively more affected by less frequent diseases. Women had a substantially higher share in spending on many highly prevalent diseases (e.g., dementia, depression, sense organ diseases and musculoskeletal disorders), while men had a higher spending share in some communicable diseases (HIV/AIDS, hepatitis), cardiovascular diseases (e.g., ischemic heart disease), mental and substance use disorders (e.g, schizophrenia, ADHD, alcohol and drug use disorders), and diabetes and chronic kidney disease.

### Methodological challenges

The biggest challenge in spending decompositions is the availability of data containing both diagnostic and spending information. Most comparable studies used encounter-level data and assigned spending to the listed diagnoses [25, 48, 49]. We did not have access to diagnostic and spending data at the level of single encounters. However, our two-step approach involving direct and regression-based assignment has two important advantages. *First*, we implicitly allowed for comorbidities for all spending types, while attributing everything to the primary diagnosis may overestimate spending for frequent main diagnoses. *Second*, we estimated coefficients for each service separately and thus allowed for different effects of diseases on the individual spending for each service. Due to very scarce diagnostic data in the outpatient sector in Switzerland, the focus was on decomposing outpatient health care spending. Our two-step spending assignment would be easily applicable to other services where no diagnostic information is collected, such as inpatient long-term care.

### Comparison with previous studies

Despite methodological differences and the different health care systems, we found similar spending shares as in the US study which applied a similar disease classification based on GBD [25]. The following comparison refers to shares of total health care spending by US public insurance (mainly Medicare and Medicaid).^5^ Our estimates were similar for mental and substance use disorders (12.0% vs. 12.9% for the US), musculoskeletal disorders (19.2% vs. 17.4%), neurological diseases (6.8% vs. 5.5%), and cardiovascular diseases (8.6% vs. 7.7%). The US estimate for diabetes (16.8%) was not directly comparable to our estimate of 2.4% as it also included urogenital, blood and other endocrine diseases. However, we would expect a higher number for the US due to the much higher prevalence of typical risk factors for diabetes, such as overweight [50]. We found higher spending shares for communicable, maternal, neonatal, and nutritional diseases (10.8% vs. 5.4%) and neoplasms (7.3% vs. 4.9%). The US study assigned 6.8% of non-injury outpatient care spending to the treatment of risk factors, which comprises conditions that we included directly in the disease groups (e.g., hypertension in cardiovascular diseases).

The only comparable study for Switzerland decomposed spending in 2011 by 21 diseases mostly consistent with the GBD level 2 conditions [11]. However, that study relied on less information to identify diseases. Our estimates were similar for musculoskeletal disorders (19.2% vs. 16.3% in [11]), mental and substance use disorders (12.0% vs. 9.5%), diabetes (2.4% vs. 2.6%), and skin and subcutaneous diseases (3.7% vs. 3.1%). We found higher shares for sense organ diseases (8.7% vs. 2.9%) and neurological disorders including dementia (6.8% vs. 2.6%) and a lower share for cardiovascular diseases (8.6% vs. 18.9%).

### Limitations

This study has some limitations. *First*, we were not able to validate our disease identification algorithms at the individual level using an external data source. Nonetheless, we compared the computed prevalence rates with the existing literature and the GBD study and performed some internal validation by running the algorithm on random subsamples.

*Second*, we did not find any diagnostic clues for some important diseases. The reasons were a *lack of specificity* of diagnostic clues (e.g., for osteoarthritis, as anti-inflammatory medication is usually unspecific [51]) and a *lack of sensitivity* (e.g., for low back pain, as claims data hold no specific diagnostic clues for the treatment of this disease). In these cases, we underestimated the true treated disease prevalence, and consequently also the spending assigned to these diseases. This limitation must be considered when comparing our results with the existing literature as in Table 2. However, this limitation applies only to some GBD level 3 diseases and less to the broader GBD level 2 major disease groups.

*Third*, a substantial part of spending (19.3%) could not be assigned to any disease. This was due to insured individuals, that could not be labelled with any condition, and to residual spending, that could not be explained by the regression model (i.e., the constant). This effect is well-known in person-based spending allocations [34]. Our algorithms were especially sensitive to acute and severe conditions. Spending for unspecific routine visits and drug prescriptions remained unexplained, especially in younger individuals.

*Fourth*, spending shares were highest for the residual ‘other’ categories which included insured who did not show any clues at the more disease-specific level 3.

*Finally*, we weighted our results both for the prevalence and the spending estimation by age and sex specific weights according to the Swiss population. They are, however, still not necessarily generalizable as the client structure of a single insurer may not be representative of the national population with respect to morbidity and geographical distribution. However, another source of information suggests that SWICA population is quite representative for the overall Swiss population: The Swiss risk equalization scheme aims to compensate for per capita spending differences due to the risk profiles of the populations enrolled with different MHI providers. Payments in the risk equalization scheme in 2017 suggest that the SWICA population was, on average, very close to the general population [22]. Moreover, the SWICA sample showed a similar age/sex structure as the Swiss population and only slightly lower per capita spending in MHI than the Swiss average.

### Future research

algorithm and, most importantly, validate it. One promising possibility to overcome the lack of diagnostic information is to link insurance claims with ICD-10 diagnoses from the Swiss inpatient registry and to check the correspondence of the diagnoses in the two sources. Furthermore, our results for the outpatient sector should be complemented with those for other health care services.

## Conclusions

At present, little is known on how much single diseases contribute to outpatient spending in Switzerland. One reason is the lack of data holding information both on spending and diseases at the individual level. Our study shows the high potential of health insurance claims data in identifying diseases when no diagnostic coding is available. Our approach may thus also be promising for epidemiological research on treated prevalence. It may be also applied in other countries, with social health insurance provided by private health insurers.

Decomposing spending by age, sex and diseases over time can inform on the drivers of health care spending. This information contributes to a better understanding of the effects of epidemiological and demographic trends on health care spending. It may be particularly important from a health policy perspective, as it can guide the definition of global spending budgets currently discussed in Switzerland and elsewhere, as well as health care provision planning.

## Supplementary Information

**Additional file 1.** Algorithms to identify diseases in the claims data.

**Additional file 2.** Additional results not shown in the article.

**Additional file 3.** Regression results.

## Data Availability

Original data are confidential and not available.

## Abbreviations

ADHD: Attention Deficit Hyperactivity Disorder
AL: Analysenliste
ATC: Anatomical-therapeutic chemical
COPD: Chronic obstructive pulmonary disease
DALY: Disability-adjusted life years
DRG: Diagnosis-related group
GBD: Global Burden of Disease
GP: General Practitioner
ICD: International Classification of Diseases
MAE: Mean absolute error
MHI: Mandatory health insurance
MiGel: Mittel- und Gegenständeliste
MRI: Magnetic resonance imaging
NCD: Non-communicable diseases
NHA: National Health Accounts
PPML: Poisson pseudo-maximum likelihood
RMSE: Root mean squared error
TarMed: Tarif médical

## Footnotes

1 USD 11,103 in 2018 with 1.18 USD/CHF exchange rate in 2018 (OECD).

2 Three service types (physiotherapy, psychiatry, dental care) were fully attributed to a disease group and no second step assignment was needed. For two services (home care and occupational therapy), we did not use the regression-based approach to assign spending in the second step.

3 We tested three other models: Ordinary least-squares (OLS) on log(y + 1), a generalized linear model (GLM) with gamma distribution and log link, and a zero-inflated negative binomial (ZINB). The PPML outperformed the other estimators in standard goodness-of-fit measures: mean absolute error (MAE), root mean squared error (RMSE) and adjusted R squared (see table 3 in the Additional file 2).

4 There were a total of 85 negative and statistically significant coefficients in the 56 models with 42 variables each (2352 coefficient estimates in total), which corresponds to a share of less than 4%.

5 We obtained these numbers from the online results tool: https://vizhub.healthdata.org/dex/
