# Supplementary material for: Decomposition of outpatient health care spending by disease - a novel approach using insurance claims data
Source: BMC Health Serv Res. 2021 Nov 22;21:1264. doi: 10.1186/s12913-021-07262-x (PMC8609863; doi:10.1186/s12913-021-07262-x)
Supplement: Supplementary file 2 — Additional file 2. Additional results not shown in the article. [file 12913_2021_7262_MOESM2_ESM.pdf]

Additional file 2

Journal: BMC Health Services Research

Article title: Decomposition of Outpatient Health Care Spending by Disease - A Novel Approach Using Insurance Claims Data

Authors: Michael Stucki
Janina Nemitz
Maria Trottmann
Simon Wieser

Corresponding author: Michael Stucki

Affiliation: Winterthur Institute of Health Economics
Zurich University of Applied Sciences

E-Mail: stcc@zhaw.ch

1 Representativeness: Descriptive statistics

	**Study sample**	**Swiss general population**
**Age/sex distribution (% of total population)**		
Female, 0-19 years	9.4%	9.7%
Female, 20-44 years	18.0%	16.5%
Female, 45-64 years	15.0%	14.0%
Female, >65 years	8.7%	10.1%
Male, 0-19 years	10.0%	10.3%
Male, 20-44 years	17.0%	17.0%
Male, 45-64 years	14.5%	14.2%
Male, >65 years	7.5%	8.1%
Total	100%	100%
**Spending**		
Total gross MHI spending (CHF, per capita, unweighted)	3461	3849
Total gross MHI spending (CHF, per capita, weighted)	3551	3849
Outpatient gross MHI spending (CHF, per capita, unweighted)	2602	2834
Outpatient gross MHI spending (CHF, per capita, weighted)	2652	2834

Table 1. Descriptive statistics of sample and comparison with Swiss general population in 2017. The weights used were the same as in the prevalence and spending estimation. Source for data on Swiss general population: age/sex distribution: *Federal Statistical Office. Population and Households Statistics (STATPOP). 2021.*; MHI spending: *Federal Office of Public Health. Statistics on the Compulsory Health Insurance 2017 (Statistik der obligatorischen Krankenversicherung 2017). 2019.* MHI=Mandatory health insurance.

2 Disease identification

	Share of patients identified by number of clues			
**Disease (level 2 (bold) and 3)**	**1**	**2**	**3**	**4**
**Communicable diseases**	**99%**	**1%**	**0%**	**0%**
HIV/AIDS	100% (Drugs)	0%	0%	0%
Other communicable diseases	99%	1%	0%	0%
Hepatitis	100% (Drugs)	0%	0%	0%
Maternal and neonatal	100% (DRG)	0%	0%	0%
Nutritional deficiencies	100% (Drugs)	0%	0%	0%
**Neoplasms**	**68%**	**24%**	**7%**	**2%**
Colon and rectum cancers	87%	13%	0%	0%
Trachea, bronchus, and lung cancers	100% (Drugs, DRG)	0%	0%	0%
Breast cancer	87%	13%	0%	0%
Prostate cancer	41%	55%	4%	0%
Other neoplasms	99%	1%	0%	0%
**Cardiovascular diseases**	**79%**	**17%**	**4%**	**0%**
Ischemic heart disease	92%	7%	1%	0%
Stroke	100% (DRG)	0%	0%	0%
Hypertensive heart disease	100% (Drugs)	0%	0%	0%
Atrial fibrillation and flutter	94%	6%	0%	0%
Other cardiovascular and circulatory diseases	100%	0%	0%	0%
**Chronic respiratory diseases**	**87%**	**12%**	**0%**	**0%**
Chronic obstructive pulmonary disease (COPD)	94%	6%	0%	0%
Asthma	100% (Drugs)	0%	0%	0%
Other chronic respiratory diseases	100%	0%	0%	0%
**Digestive diseases**	**91%**	**7%**	**1%**	**0%**
Cirrhosis and other chronic liver diseases	99%	1%	0%	0%
Other digestive diseases	97%	3%	0%	0%
**Neurological disorders**	**91%**	**9%**	**0%**	**0%**
Alzheimer's disease and other dementias	100% (Drugs)	0%	0%	0%
Parkinson's disease	100% (Drugs)	0%	0%	0%
Epilepsy	99%	1%	0%	0%
Multiple sclerosis	98%	2%	0%	0%
Other neurological diseases	100%	0%	0%	0%
**Mental and substance use disorders**	**62%**	**33%**	**5%**	**0%**
Schizophrenia	100% (Drugs)	0%	0%	0%
Depression	100% (Drugs)	0%	0%	0%
Attention Deficit Hyperactivity Disorder (ADHD)	100% (Drugs)	0%	0%	0%
Alcohol and drug use disorders	89%	11%	0%	0%
Other mental disorders	100%	0%	0%	0%
**Diabetes and kidney disease**	**93%**	**7%**	**1%**	**0%**
Diabetes mellitus	99%	1%	0%	0%
Chronic kidney disease	100% (Drugs)	0%	0%	0%
**Skin and subcutaneous diseases**	**77%**	**21%**	**2%**	**0%**
**Sense organ diseases**	**62%**	**33%**	**5%**	**0%**
**Musculoskeletal disorders**	**70%**	**28%**	**3%**	**0%**
Rheumatoid arthritis	100% (Drugs)	0%	0%	0%
Osteoarthritis	100% (Drugs, DRG)	0%	0%	0%
Low back pain	100% (Tariff cat.)	0%	0%	0%
Osteoporosis	100% (Drugs)	0%	0%	0%
Other musculoskeletal disorders	100%	0%	0%	0%
**Other non-communicable diseases**	**96%**	**4%**	**0%**	**0%**
Oral disorders	99%	1%	0%	0%
Other non-communicable diseases	97%	3%	0%	0%
**Well care**	**100%** (Financing, drugs, DRG)	**0%**	**0%**	**0%**

Table 2. Share of patients by disease identified by the number of clues, for each disease at GBD level 2 and level 3 (in brackets: the type of clue used when 100% of the patients were identified based on only one type of clue)

3 Model selection

Service	Measure	OLS log(y+1)	GLM (gamma, log link)	PPML	ZINB
Physician (GP)	Adjusted R squared	0	0	4	4
	Mean absolute error	0	0	2	6
	Root mean squared error	0	0	4	4
Physician (specialist)	Adjusted R squared	0	0	1	7
	Mean absolute error	5	0	2	1
	Root mean squared error	0	0	8	0
Hospital outpatient	Adjusted R squared	0	0	7	1
	Mean absolute error	0	0	8	0
	Root mean squared error	0	0	8	0
Drugs	Adjusted R squared	0	0	7	1
	Mean absolute error	0	0	8	0
	Root mean squared error	0	0	8	0
Laboratory	Adjusted R squared	0	0	6	2
	Mean absolute error	0	0	6	2
	Root mean squared error	0	0	5	3
Radiology	Adjusted R squared	0	0	7	1
	Mean absolute error	2	0	4	2
	Root mean squared error	0	0	8	0
Other outpatient	Adjusted R squared	0	0	5	3
	Mean absolute error	0	0	6	2
	Root mean squared error	0	0	6	2
All services		7	0	120	41

Table 3. Comparison of models for regression-based spending assignment. Table shows the number of times in which one of the three estimators performed best according to three measures (Adj. R2, mean absolute error, and root mean squared error). The adjusted R squared was defined as 1-[(1-R2)*(n-1)/dof] (dof=degrees of freedom) and with R2 defined as the correlation between the observed and the predicted value on the original scale. We estimated a total of 56 models, resulting in 168 measures for model selection. The 56 models were estimated for 7 services, 4 age categories, and by sex. For 15 out of 56 models, convergence was not achieved with the ZINB models. In these cases, the best estimator was chosen among the three remaining estimators. PPML=Poisson pseudo maximum likelihood. ZINB=Zero-inflated negative binomial

4 Spending by disease and sex

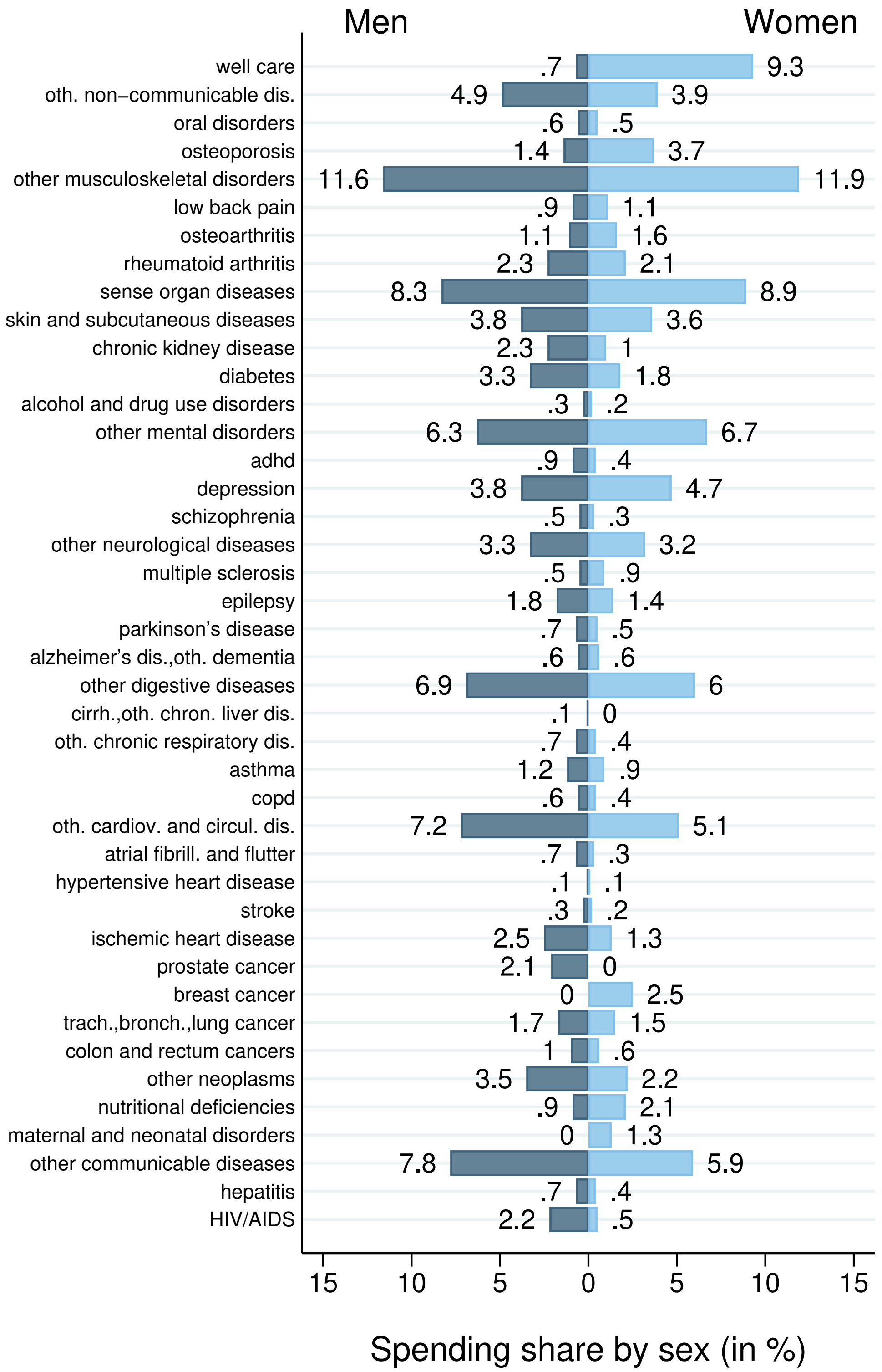

Figure 1. Outpatient spending by disease (GBD level 3; % of total assigned spending), for men and women separately

5 Spending by disease and age

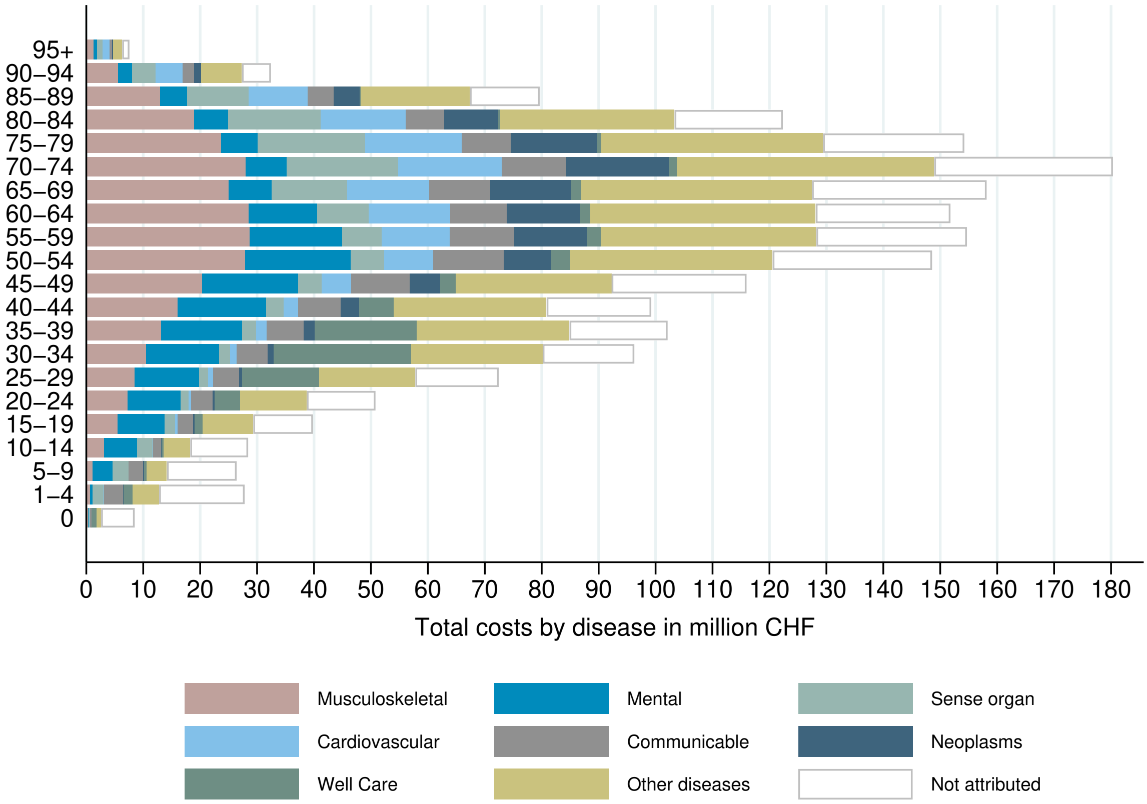

Figure 2. Outpatient spending by age groups and major disease groups, in m CHF

6 Spending by disease and service

Disease	Total outpatient	Physician (GP)	Physician (specialist)	Hospital outpatient	Drugs outpatient	Psychiatry outpatient	Home care	Laboratory	Radiology	Physiotherapy	Occ. therapy	Dental care outpatient	Other outpatient
HIV/AIDS	1.2	0.0	0.0	0.3	3.4	0.0	0.0	1.6	0.1	0.0	0.0	0.0	0.6
hepatitis	0.5	0.0	0.0	0.1	1.7	0.0	0.0	0.2	0.0	0.0	0.0	0.0	0.1
other communicable diseases	6.7	13.8	4.2	11.5	3.2	0.0	8.6	17.1	9.1	0.0	0.0	0.0	9.3
maternal and neonatal disorders	0.8	0.1	1.8	3.2	0.0	0.0	0.0	1.0	0.0	0.0	0.0	0.0	0.8
nutritional deficiencies	1.6	3.8	0.2	0.9	1.8	0.0	2.1	5.4	0.9	0.0	0.0	0.0	2.6
other neoplasms	2.7	0.1	1.0	5.2	5.0	0.0	0.7	2.6	2.2	0.0	0.0	0.0	1.7
colon and rectum cancers	0.7	0.1	0.6	1.4	1.0	0.0	0.3	0.9	1.2	0.0	0.0	0.0	0.3
trachea, bronchus, and lung cancers	1.6	0.0	0.4	1.4	4.2	0.0	0.1	0.6	0.8	0.0	0.0	0.0	0.1
breast cancer	1.4	0.1	0.6	3.6	2.5	0.0	0.2	0.8	1.3	0.0	0.0	0.0	0.6
prostate cancer	0.9	0.1	0.4	1.7	1.7	0.0	0.2	0.5	0.9	0.0	0.0	0.0	0.0
ischemic heart disease	1.8	2.6	1.9	3.1	1.6	0.0	1.4	1.9	1.8	0.0	0.0	0.0	2.4
stroke	0.2	0.5	0.1	0.4	0.2	0.0	0.8	0.2	0.2	0.0	0.0	0.0	0.3
hypertensive heart disease	0.1	0.0	0.0	0.1	0.3	0.0	0.1	0.1	0.0	0.0	0.0	0.0	0.1
atrial fibrillation and flutter	0.5	0.6	0.5	0.6	0.6	0.0	0.5	0.4	0.1	0.0	0.0	0.0	0.5
other cardiovascular and circulatory diseases	6.0	8.4	6.4	3.5	8.7	0.0	12.0	4.1	4.2	0.0	0.0	0.0	6.1
copd	0.5	0.1	0.2	0.1	0.9	0.0	1.0	0.1	0.1	0.0	0.0	0.0	1.3
asthma	1.0	0.8	1.2	0.3	2.0	0.0	1.4	0.5	0.2	0.0	0.0	0.0	0.2
other chronic respiratory diseases	0.5	0.1	1.2	0.1	0.6	0.0	0.4	0.4	0.4	0.0	0.0	0.0	0.9
cirrhosis and other chronic liver diseases	0.0	0.0	0.0	0.0	0.0	0.0	0.0	0.2	0.1	0.0	0.0	0.0	0.0
other digestive diseases	6.4	5.9	10.5	8.9	6.6	0.0	3.5	10.3	9.8	0.0	0.0	0.0	1.2
alzheimer's disease and other dementias	0.6	0.8	0.4	0.1	1.0	0.0	2.5	0.2	0.5	0.0	3.5	0.0	0.4
parkinson's disease	0.6	0.5	0.3	0.2	1.2	0.0	1.6	0.0	0.3	0.0	2.9	0.0	0.7
epilepsy	1.6	1.4	0.7	2.2	2.2	0.0	2.2	1.3	1.8	0.0	6.8	0.0	1.9
multiple sclerosis	0.8	0.0	0.2	0.2	2.3	0.0	0.1	0.1	0.5	0.0	0.5	0.0	0.1
other neurological diseases	3.3	4.4	3.4	5.1	2.7	0.0	3.6	2.7	7.1	0.0	20.1	0.0	3.2
schizophrenia	0.3	0.1	0.0	0.0	0.6	1.9	0.4	0.1	0.0	0.0	0.0	0.0	0.2
depression	4.3	4.3	0.1	1.0	3.9	31.3	5.0	1.7	0.7	0.0	0.0	0.0	4.7
adhd	0.6	0.2	0.1	0.1	0.7	5.3	0.1	0.1	0.0	0.0	0.0	0.0	0.3
other mental disorders	6.5	4.5	0.1	2.1	3.9	60.3	5.7	2.4	0.6	0.0	0.0	0.0	6.4
alcohol and drug use disorders	0.2	0.2	0.0	0.1	0.3	1.2	0.2	0.1	0.0	0.0	0.0	0.0	0.4
diabetes	2.4	1.3	0.5	0.9	4.4	0.0	3.6	1.7	0.0	0.0	0.0	0.0	7.2
chronic kidney disease	1.6	0.0	0.0	0.3	0.6	0.0	0.3	0.5	0.2	0.0	0.0	0.0	16.7
skin and subcutaneous diseases	3.7	4.1	10.6	1.4	2.9	0.0	6.9	5.6	1.8	0.0	0.0	0.0	1.8
sense organ diseases	8.7	7.1	29.9	6.4	5.8	0.0	9.1	4.2	4.4	0.0	0.0	0.0	8.8
rheumatoid arthritis	2.2	0.2	0.5	0.6	5.8	0.0	0.3	1.6	0.9	1.5	1.6	0.0	0.3
osteoarthritis	1.4	1.8	1.1	0.2	1.0	0.0	0.7	0.8	3.4	8.1	7.5	0.0	0.1
low back pain	1.0	1.2	1.4	1.4	0.3	0.0	0.3	0.2	3.4	4.0	2.5	0.0	0.3
other musculoskeletal disorders	11.8	17.8	5.5	5.8	4.7	0.0	10.8	8.2	24.1	76.0	46.5	0.0	5.7
osteoporosis	2.7	2.7	0.8	1.5	3.5	0.0	4.3	2.5	3.0	10.2	8.2	0.0	0.1
oral disorders	0.6	0.6	0.3	0.5	0.3	0.0	0.8	0.6	0.3	0.0	0.0	92.8	3.0
other non−communicable diseases	4.3	3.2	2.6	4.0	6.2	0.0	7.0	8.4	6.8	0.0	0.0	0.0	1.7
well care	5.7	6.4	10.3	19.5	0.1	0.0	1.0	7.8	6.8	0.2	0.0	7.2	6.9

100

0

Figure 3. Outpatient spending shares by disease (GBD level 3) and service (in % of total assigned spending)

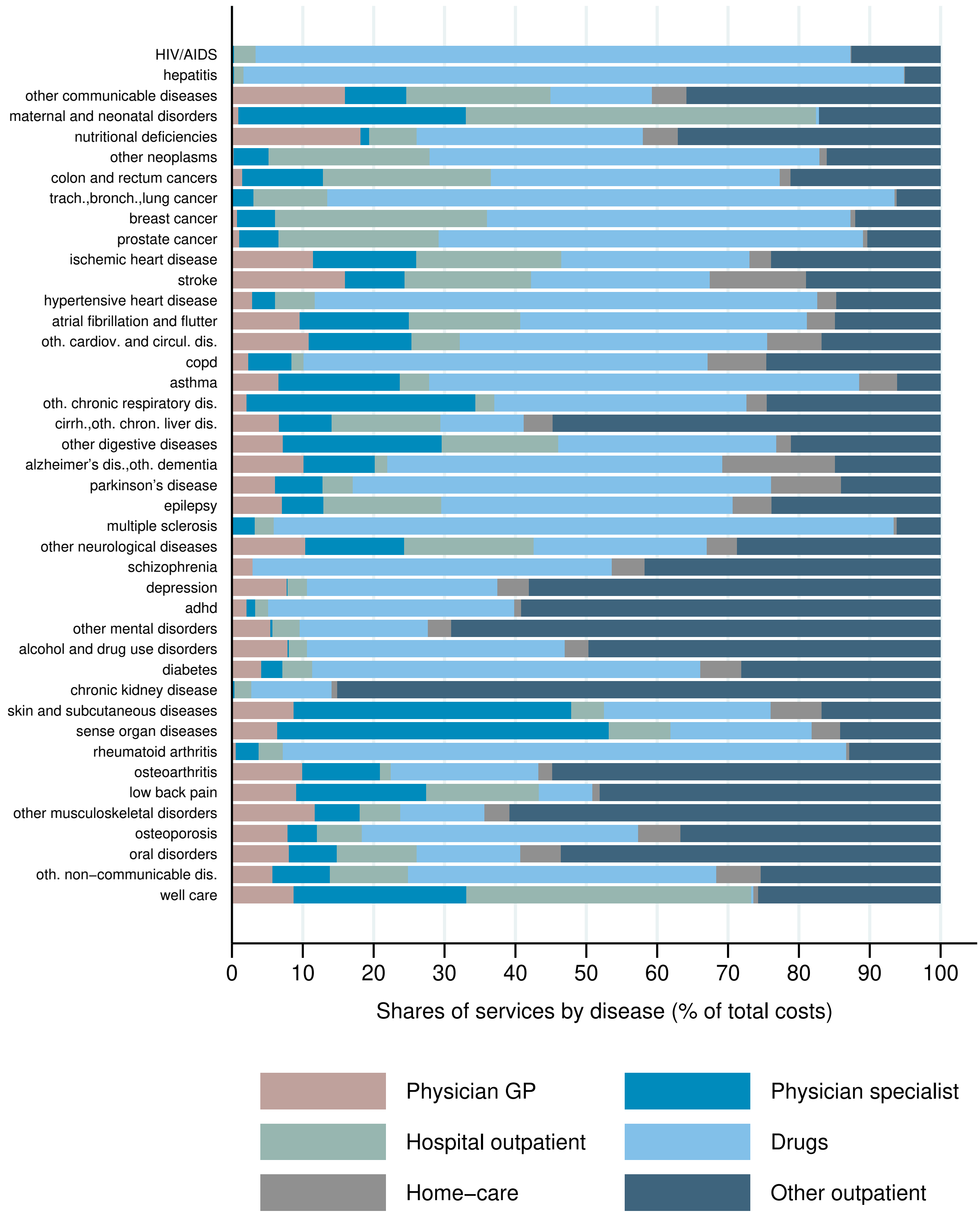

Figure 4. Spending shares of outpatient services by disease (GBD level 3). GP: General practitioner. Note: the category "other outpatient" comprises all the outpatient services not explicitly shown in the graph (physiotherapy, occupational therapy, psychotherapy, radiology, laboratory tests, dental care, other outpatient)

7 Scenario Analysis

**Disease (GBD level 2)**	**Minimum**	**Maximum**	**Mean**
Musculoskeletal disorders	16.6	21.0	19.0
Mental and substance use disorders	12.0	13.7	12.7
Cardiovascular diseases	8.5	11.2	9.8
Sense organ diseases	7.9	9.0	8.3
Neoplasms	7.3	9.1	8.1
Communicable diseases	5.3	9.6	7.3
Well care	5.7	7.6	6.5
Neurological disorders	5.5	7.0	6.1
Digestive diseases	5.4	6.5	6.0
Other non-communicable diseases	4.2	5.6	4.9
Diabetes and kidney diseases	4.0	4.7	4.3
Skin and subcutaneous diseases	1.8	3.7	2.8
Chronic respiratory diseases	1.8	2.5	2.2
Nutritional deficiencies	0.5	1.9	1.1
Maternal and neonatal disorders	0.8	0.9	0.8

Table 4. minimum, maximum, and mean spending share resulting from scenario analysis (GBD level 2). The scenarios include different combinations of minimum requirements of drug utilization (number of packages) and spending at a physician with a certain specialization used in the identification of diseases.

Disease (GBD level 3)	Minimum	Maximum	Mean
HIV/AIDS	1.2	1.4	1.3
adhd	.6	.7	.6
alcohol and drug use disorders	.2	.2	.2
alzheimer's disease and other dementias	.4	.7	.5
asthma	1	1.6	1.2
atrial fibrillation and flutter	.4	.5	.5
breast cancer	1.4	1.7	1.6
chronic kidney disease	1.5	1.8	1.6
cirrhosis and other chronic liver diseases	0	.1	0
colon and rectum cancers	.7	1	.8
congenital birth defects	0	0	0
copd	.5	.6	.5
depression	4.3	5.4	4.7
diabetes	2.4	2.9	2.6
epilepsy	1.6	2.3	1.9
hepatitis	.5	.6	.5
hypertensive heart disease	.1	.1	.1
ischemic heart disease	1.8	2.3	2
low back pain	1	1.5	1.2
maternal and neonatal disorders	.8	.9	.8
multiple sclerosis	.7	.8	.8
nutritional deficiencies	.5	1.9	1.1
oral disorders	.3	.6	.5
osteoarthritis	.7	1.5	1.1
osteoporosis	2.7	3.4	3.1
other cardiovascular and circulatory diseases	5.8	7.9	6.9
other chronic respiratory diseases	.3	.6	.4
other communicable diseases	3.6	7.8	5.5
other digestive diseases	5.4	6.5	6
other mental disorders	6.4	7.3	6.8
other musculoskeletal disorders	8.6	13.5	11.3
other neoplasms	2.7	3.6	3.1
other neurological diseases	1.3	3.3	2.3
other non-communicable diseases	3.9	5.2	4.5
parkinson's disease	.6	.7	.6
prostate cancer	.9	1	.9
rheumatoid arthritis	2.2	2.5	2.3
schizophrenia	.3	.4	.3
sense organ diseases	7.9	9	8.3
skin and subcutaneous diseases	1.8	3.7	2.8
stroke	.2	.4	.3
trachea, bronchus, and lung cancers	1.6	1.8	1.7
well care	5.7	7.6	6.5

Table 5. minimum, maximum, and mean spending share resulting from scenario analysis (GBD level 3). The scenarios include different combinations of minimum requirements of drug utilization (number of packages) and spending at a physician with a certain specialization used in the identification of diseases.
