# Supplementary material for: Decomposition of outpatient health care spending by disease - a novel approach using insurance claims data
Source: BMC Health Serv Res. 2021 Nov 22;21:1264. doi: 10.1186/s12913-021-07262-x (PMC8609863; doi:10.1186/s12913-021-07262-x)
Supplement: Supplementary file 3 — Additional file 3. Regression results. [file 12913_2021_7262_MOESM3_ESM.pdf]

Additional file 3

Journal: BMC Health Services Research

Article title: Decomposition of Outpatient Health Care Spending by Disease - A Novel Approach Using Insurance Claims Data

Authors: Michael Stucki
Janina Nemitz
Maria Trottmann
Simon Wieser

Corresponding author: Michael Stucki

Affiliation: Winterthur Institute of Health Economics
Zurich University of Applied Sciences

E-Mail: stcc@zhaw.ch

1 Regression output: Physician (general practitioners)

TABLE 1. REGRESSION COEFFICIENTS AND STANDARD ERRORS FROM POISSON PSEUDO-MAXIMUM LIKELIHOOD MODELS

	0-19 y. (f)	0-19 y. (m)	20-44 y. (f)	20-44 y. (m)	45-64 y. (f)	45-64 y. (m)	65+ y. (f)	65+ y. (m)
HIV/AIDS	-0.942*	-1.214	-0.025	0.280*	-0.044	0.136	-0.095	-0.124
	(0.221)	(0.657)	(0.131)	(0.109)	(0.095)	(0.084)	(0.202)	(0.102)
other communicable diseases	0.760*	0.728*	0.482*	0.695*	0.368*	0.461*	0.364*	0.371*
	(0.022)	(0.024)	(0.010)	(0.015)	(0.011)	(0.014)	(0.011)	(0.013)
hepatitis			0.897*	0.262	0.195	0.292*	-0.033	-0.077
			(0.353)	(0.258)	(0.136)	(0.130)	(0.246)	(0.165)
maternal and neonatal disorders	0.342	0.055	-0.550*		-0.554			
	(0.396)	(0.548)	(0.047)		(0.317)			
nutritional deficiencies	0.978*	0.872*	0.490*	0.410*	0.465*	0.325*	0.266*	0.348*
	(0.028)	(0.050)	(0.012)	(0.068)	(0.015)	(0.046)	(0.017)	(0.027)
colon and rectum cancers	-0.253		0.648*	0.520	0.176	0.425*	0.136*	0.202*
	(1.225)		(0.190)	(0.509)	(0.092)	(0.145)	(0.055)	(0.093)
trachea, bronchus, and lung cancers	-0.720		-0.279	-1.085*	-0.163	0.002	-0.087	-0.140
	(1.559)		(0.235)	(0.434)	(0.115)	(0.188)	(0.086)	(0.075)
breast cancer			0.187		0.299*		0.088*	
			(0.125)		(0.064)		(0.041)	
prostate cancer		0.826*		0.736		0.271*		0.187*
		(0.337)		(0.571)		(0.130)		(0.041)
other neoplasms	0.048	-0.174	-0.113	0.036	0.032	0.052	0.019	0.133*
	(0.185)	(0.210)	(0.098)	(0.131)	(0.049)	(0.064)	(0.029)	(0.035)
ischemic heart disease	0.674*	0.717*	0.327*	0.438*	0.387*	0.560*	0.521*	0.443*
	(0.120)	(0.130)	(0.037)	(0.046)	(0.024)	(0.027)	(0.020)	(0.020)
stroke		0.305*	-0.185	0.489*	0.566*	0.512*	0.488*	0.443*
		(0.015)	(0.280)	(0.209)	(0.123)	(0.108)	(0.035)	(0.035)
hypertensive heart disease			0.591	0.408	0.161	0.440*	0.201*	0.305*
			(0.436)	(0.266)	(0.178)	(0.096)	(0.072)	(0.088)
atrial fibrillation and flutter	0.550	0.949*	0.501	0.024	0.246*	0.456*	0.452*	0.397*
	(0.448)	(0.399)	(0.310)	(0.202)	(0.092)	(0.071)	(0.039)	(0.031)
other cardiovascular and circulatory diseases	0.375*	0.445*	0.185*	0.478*	0.301*	0.408*	0.302*	0.280*
	(0.098)	(0.080)	(0.020)	(0.031)	(0.013)	(0.014)	(0.011)	(0.014)

Table 1. Regression coefficients and standard errors from Poisson pseudo-maximum likelihood models

	0-19 y. (f)	0-19 y. (m)	20-44 y. (f)	20-44 y. (m)	45-64 y. (f)	45-64 y. (m)	65+ y. (f)	65+ y. (m)
copd	0.049	0.339	-0.254*	-0.329	0.097*	0.096	0.101*	0.055*
	(0.247)	(0.244)	(0.114)	(0.180)	(0.042)	(0.049)	(0.026)	(0.025)
asthma	0.364*	0.444*	0.279*	0.338*	0.124*	0.187*	0.073*	0.116*
	(0.055)	(0.048)	(0.028)	(0.036)	(0.021)	(0.028)	(0.018)	(0.022)
other chronic respiratory diseases	0.663*	0.563*	0.255*	0.195*	0.120*	0.167*	-0.005	0.030
	(0.180)	(0.148)	(0.055)	(0.072)	(0.039)	(0.039)	(0.036)	(0.039)
cirrhosis and other chronic liver diseases			0.234	0.957*	0.339	0.612*	0.229	0.591*
			(0.338)	(0.322)	(0.181)	(0.166)	(0.164)	(0.143)
other digestive diseases	0.438*	0.413*	0.429*	0.521*	0.355*	0.386*	0.224*	0.222*
	(0.044)	(0.044)	(0.015)	(0.021)	(0.012)	(0.014)	(0.012)	(0.013)
alzheimer's disease and other dementias	0.330	0.535	0.418*	0.315*	0.341*	0.330*	0.210*	0.245*
	(0.186)	(0.279)	(0.070)	(0.116)	(0.040)	(0.045)	(0.018)	(0.022)
parkinson's disease	0.339	0.469	0.419*	0.183	0.299*	0.306*	0.244*	0.317*
	(0.327)	(0.298)	(0.088)	(0.147)	(0.064)	(0.064)	(0.025)	(0.034)
epilepsy	0.371*	0.127	0.526*	0.654*	0.354*	0.439*	0.307*	0.257*
	(0.117)	(0.157)	(0.042)	(0.050)	(0.029)	(0.039)	(0.020)	(0.023)
multiple sclerosis	1.563*	0.396*	0.267	0.539*	0.132	0.079	-0.018	0.189
	(0.623)	(0.026)	(0.136)	(0.215)	(0.078)	(0.155)	(0.166)	(0.195)
other neurological diseases	0.467*	0.530*	0.393*	0.498*	0.325*	0.399*	0.243*	0.245*
	(0.057)	(0.062)	(0.015)	(0.021)	(0.013)	(0.019)	(0.016)	(0.017)
schizophrenia	1.721*	0.710	0.287*	0.327*	0.302*	0.314*	0.362*	0.279*
	(0.521)	(0.462)	(0.084)	(0.095)	(0.080)	(0.090)	(0.108)	(0.074)
depression	0.909*	1.038*	0.658*	0.781*	0.418*	0.480*	0.336*	0.315*
	(0.076)	(0.103)	(0.016)	(0.027)	(0.014)	(0.020)	(0.014)	(0.018)
adhd	0.263*	0.638*	0.308*	0.352*	-0.050	0.128	-0.190	0.226
	(0.117)	(0.066)	(0.054)	(0.061)	(0.060)	(0.095)	(0.147)	(0.164)
other mental disorders	0.604*	0.614*	0.541*	0.600*	0.387*	0.370*	0.326*	0.306*
	(0.037)	(0.041)	(0.017)	(0.025)	(0.017)	(0.026)	(0.017)	(0.020)
alcohol and drug use disorders	-0.154	1.042*	0.368*	0.612*	0.384*	0.600*	0.166	0.253*
	(0.516)	(0.312)	(0.120)	(0.083)	(0.091)	(0.058)	(0.103)	(0.084)
diabetes	0.171	0.525*	0.196*	0.290*	0.194*	0.276*	0.108*	0.166*
	(0.156)	(0.165)	(0.042)	(0.069)	(0.021)	(0.023)	(0.015)	(0.015)
chronic kidney disease		0.638	-0.790*	-1.579*	-0.537*	-0.471*	0.047	-0.067
		(0.417)	(0.380)	(0.457)	(0.259)	(0.195)	(0.080)	(0.071)

Table 1. Regression coefficients and standard errors from Poisson pseudo-maximum likelihood models

	0-19 y. (f)	0-19 y. (m)	20-44 y. (f)	20-44 y. (m)	45-64 y. (f)	45-64 y. (m)	65+ y. (f)	65+ y. (m)
skin and subcutaneous diseases	0.301*	0.358*	0.214*	0.353*	0.166*	0.217*	0.154*	0.164*
	(0.024)	(0.026)	(0.011)	(0.018)	(0.012)	(0.015)	(0.011)	(0.012)
sense organ diseases	0.155*	0.218*	0.288*	0.339*	0.203*	0.246*	0.188*	0.190*
	(0.023)	(0.024)	(0.013)	(0.019)	(0.010)	(0.013)	(0.011)	(0.011)
rheumatoid arthritis	0.146	0.641	0.352*	0.320*	0.261*	0.086	0.100*	0.053
	(0.212)	(0.402)	(0.075)	(0.080)	(0.047)	(0.056)	(0.036)	(0.048)
osteoarthritis	1.072*	1.407*	0.585*	0.741*	0.509*	0.576*	0.210*	0.243*
	(0.107)	(0.129)	(0.063)	(0.088)	(0.020)	(0.033)	(0.017)	(0.022)
low back pain	0.912*	0.875*	0.785*	0.887*	0.692*	0.634*	0.377*	0.356*
	(0.169)	(0.195)	(0.064)	(0.085)	(0.038)	(0.054)	(0.029)	(0.036)
osteoporosis	0.623*	0.774	0.493*	0.269*	0.426*	0.298*	0.356*	0.315*
	(0.250)	(0.537)	(0.059)	(0.103)	(0.022)	(0.051)	(0.014)	(0.025)
other musculoskeletal disorders	0.699*	0.791*	0.632*	0.811*	0.578*	0.572*	0.364*	0.340*
	(0.024)	(0.025)	(0.011)	(0.016)	(0.011)	(0.013)	(0.012)	(0.012)
oral disorders	0.325*	0.273*	0.223*	0.273*	0.238*	0.112*	0.080*	0.114*
	(0.044)	(0.048)	(0.027)	(0.035)	(0.030)	(0.034)	(0.022)	(0.029)
other non-communicable diseases	0.278*	0.308*	0.154*	0.271*	0.191*	0.244*	0.084*	0.139*
	(0.072)	(0.084)	(0.017)	(0.036)	(0.012)	(0.016)	(0.011)	(0.012)
well care	0.198*	-0.406*	-0.046*	-1.447*	0.034*	-0.879*	-0.069*	-0.349*
	(0.027)	(0.050)	(0.010)	(0.056)	(0.010)	(0.049)	(0.012)	(0.054)
Constant	3.607*	3.575*	4.458*	4.076*	4.723*	4.593*	5.275*	5.240*
	(0.015)	(0.015)	(0.008)	(0.009)	(0.009)	(0.009)	(0.012)	(0.013)
Adjusted R^2	0.13	0.10	0.23	0.22	0.23	0.21	0.20	0.20
Observations	66567	71059	127532	120801	106368	102632	61884	52945

b coefficients; se in parentheses; f: female, m:male

* $p < 0.05$

The adj. R2 was defined as 1-[(1-R2)*(n-1)/dof]; R2 was defined as the correlation between observed and predicted values on the original scale

2 Regression output: Physician (specialists)

Table 2. Regression coefficients and standard errors from Poisson pseudo-maximum likelihood models

	0-19 y. (f)	0-19 y. (m)	20-44 y. (f)	20-44 y. (m)	45-64 y. (f)	45-64 y. (m)	65+ y. (f)	65+ y. (m)
HIV/AIDS	-0.127	-0.885*	-0.987*	0.274	-0.074	-0.117	0.372	0.082
	(0.365)	(0.364)	(0.329)	(0.213)	(0.239)	(0.179)	(0.554)	(0.184)
other communicable diseases	0.643*	0.670*	0.542*	0.840*	0.550*	0.808*	0.496*	0.664*
	(0.014)	(0.014)	(0.026)	(0.043)	(0.035)	(0.033)	(0.033)	(0.029)
hepatitis			-0.443	-1.796*	-0.643	-0.543	-0.856	-0.235
			(0.554)	(0.591)	(0.358)	(0.399)	(0.550)	(0.594)
maternal and neonatal disorders	1.251*	0.832*	-0.148		-0.969			
	(0.159)	(0.160)	(0.081)		(0.536)			
nutritional deficiencies	0.018	0.436*	0.121*	0.111	0.174*	0.147	0.023	0.085
	(0.031)	(0.031)	(0.033)	(0.228)	(0.042)	(0.109)	(0.055)	(0.071)
colon and rectum cancers	-1.754*		0.328	0.104	0.431*	0.143	0.284*	-0.134
	(0.547)		(0.305)	(0.387)	(0.178)	(0.225)	(0.120)	(0.120)
trachea, bronchus, and lung cancers		-0.323	-0.461	-2.594*	-0.376	-1.058*	0.041	-0.011
		(0.296)	(0.310)	(0.532)	(0.207)	(0.248)	(0.189)	(0.145)
breast cancer			-0.039		0.290*		0.135	
			(0.182)		(0.093)		(0.081)	
prostate cancer		-0.192		1.639*		0.874*		0.875*
		(0.450)		(0.269)		(0.112)		(0.049)
other neoplasms	-0.143	-0.309	0.299*	-0.079	0.106	0.241*	0.106	0.327*
	(0.202)	(0.160)	(0.122)	(0.157)	(0.089)	(0.097)	(0.066)	(0.060)
ischemic heart disease	-0.567*	-0.699*	0.113	0.225	0.094	-0.029	0.154*	0.175*
	(0.142)	(0.147)	(0.092)	(0.167)	(0.090)	(0.070)	(0.060)	(0.052)
stroke	1.956*	0.127*	-0.732	-0.561	-0.170	-0.072	-0.278*	-0.049
	(0.009)	(0.008)	(0.668)	(0.909)	(0.270)	(0.214)	(0.117)	(0.100)
hypertensive heart disease			-1.853	0.414	0.078	-0.063	-0.191	0.449*
			(1.106)	(0.405)	(0.247)	(0.226)	(0.229)	(0.190)
atrial fibrillation and flutter	-1.552*	0.032	0.831*	0.313	0.037	0.437*	0.018	0.102
	(0.322)	(0.221)	(0.278)	(0.421)	(0.159)	(0.169)	(0.086)	(0.079)
other cardiovascular and circulatory diseases	0.129	-0.097	0.457*	0.372*	0.212*	0.179*	0.085*	0.054
	(0.099)	(0.076)	(0.051)	(0.081)	(0.051)	(0.038)	(0.032)	(0.029)

Table 2. Regression coefficients and standard errors from Poisson pseudo-maximum likelihood models

	0-19 y. (f)	0-19 y. (m)	20-44 y. (f)	20-44 y. (m)	45-64 y. (f)	45-64 y. (m)	65+ y. (f)	65+ y. (m)
copd	0.030	0.309	0.667	-0.645*	-0.168	-0.279	-0.047	-0.117
	(0.226)	(0.249)	(0.601)	(0.316)	(0.122)	(0.168)	(0.089)	(0.072)
asthma	0.685*	0.635*	0.139*	0.407*	0.168*	0.139	-0.020	0.004
	(0.029)	(0.023)	(0.063)	(0.081)	(0.061)	(0.080)	(0.054)	(0.057)
other chronic respiratory diseases	0.300*	0.371*	0.589*	0.842*	0.390*	0.457*	0.209*	0.210*
	(0.141)	(0.123)	(0.088)	(0.112)	(0.072)	(0.082)	(0.077)	(0.060)
cirrhosis and other chronic liver diseases			-1.229	0.895	-0.213	-0.169	-0.889	-0.439
			(0.653)	(0.515)	(0.445)	(0.324)	(0.539)	(0.316)
other digestive diseases	0.621*	0.611*	0.751*	1.106*	0.570*	0.679*	0.483*	0.347*
	(0.027)	(0.021)	(0.038)	(0.054)	(0.045)	(0.034)	(0.034)	(0.030)
alzheimer's disease and other dementias	-0.200	0.326	0.991*	0.950*	0.895*	0.954*	0.371*	0.382*
	(0.279)	(0.241)	(0.093)	(0.133)	(0.055)	(0.099)	(0.052)	(0.055)
parkinson's disease	-0.578	-0.730	0.217	-0.741*	0.188	0.185	0.159*	0.146
	(0.829)	(0.572)	(0.223)	(0.278)	(0.123)	(0.138)	(0.074)	(0.092)
epilepsy	0.229*	0.312*	0.538*	0.646*	0.443*	0.356*	0.253*	0.146*
	(0.095)	(0.096)	(0.099)	(0.160)	(0.082)	(0.079)	(0.052)	(0.052)
multiple sclerosis	-15.958*		-0.267	-0.798*	0.176	0.534	0.565	-0.029
	(1.006)		(0.207)	(0.294)	(0.198)	(0.434)	(0.381)	(0.655)
other neurological diseases	0.179*	0.179*	0.418*	0.336*	0.551*	0.417*	0.530*	0.469*
	(0.057)	(0.049)	(0.041)	(0.073)	(0.056)	(0.044)	(0.040)	(0.037)
schizophrenia	-2.470*	-0.788	-0.387	0.092	-0.469*	-0.156	-0.132	-0.512
	(1.000)	(0.658)	(0.225)	(0.207)	(0.178)	(0.192)	(0.213)	(0.274)
depression	-0.497*	-0.641*	0.213*	0.272*	0.072	0.136*	-0.061	0.032
	(0.095)	(0.152)	(0.048)	(0.081)	(0.039)	(0.059)	(0.041)	(0.053)
adhd	0.395*	0.307*	-0.031	0.194	0.236	-0.404	0.090	0.131
	(0.074)	(0.046)	(0.108)	(0.114)	(0.146)	(0.263)	(0.377)	(0.354)
other mental disorders	0.134*	0.130*	0.193*	0.264*	0.052	0.182*	-0.058	-0.171*
	(0.035)	(0.032)	(0.049)	(0.074)	(0.060)	(0.057)	(0.075)	(0.051)
alcohol and drug use disorders	-0.016	-0.701	0.478	0.467*	-0.046	0.052	-0.016	0.272
	(0.262)	(0.407)	(0.286)	(0.234)	(0.192)	(0.154)	(0.269)	(0.241)
diabetes	0.331*	0.463*	-0.248*	-0.282	-0.242*	-0.322*	-0.180*	-0.133*
	(0.151)	(0.168)	(0.107)	(0.170)	(0.058)	(0.070)	(0.051)	(0.038)
chronic kidney disease		-0.313	0.116	-1.654*	-0.705	-0.249	-0.179	-0.325*
		(0.641)	(0.352)	(0.663)	(0.375)	(0.353)	(0.135)	(0.158)

Table 2. Regression coefficients and standard errors from Poisson pseudo-maximum likelihood models

	0-19 y. (f)	0-19 y. (m)	20-44 y. (f)	20-44 y. (m)	45-64 y. (f)	45-64 y. (m)	65+ y. (f)	65+ y. (m)
skin and subcutaneous diseases	0.189*	0.286*	0.377*	0.592*	0.350*	0.409*	0.379*	0.423*
	(0.016)	(0.016)	(0.031)	(0.046)	(0.037)	(0.034)	(0.039)	(0.028)
sense organ diseases	0.366*	0.388*	0.555*	0.763*	0.433*	0.506*	0.602*	0.460*
	(0.014)	(0.014)	(0.030)	(0.043)	(0.033)	(0.031)	(0.035)	(0.028)
rheumatoid arthritis	-0.292	-0.002	0.126	0.043	0.108	0.168	-0.021	-0.060
	(0.191)	(0.246)	(0.168)	(0.199)	(0.095)	(0.143)	(0.091)	(0.089)
osteoarthritis	-0.655*	-1.117*	0.183	0.327	0.312*	0.307*	0.325*	0.210*
	(0.244)	(0.332)	(0.130)	(0.225)	(0.057)	(0.079)	(0.079)	(0.051)
low back pain	-0.194	-0.115	1.376*	1.471*	1.456*	1.243*	0.954*	0.724*
	(0.183)	(0.186)	(0.127)	(0.153)	(0.140)	(0.093)	(0.114)	(0.069)
osteoporosis	-0.139	0.561*	0.581*	-0.199	0.228*	0.172	0.160*	-0.011
	(0.372)	(0.211)	(0.192)	(0.258)	(0.057)	(0.119)	(0.044)	(0.060)
other musculoskeletal disorders	-0.072*	-0.071*	0.416*	0.341*	0.407*	0.349*	0.173*	0.151*
	(0.020)	(0.021)	(0.030)	(0.048)	(0.030)	(0.033)	(0.032)	(0.029)
oral disorders	0.489*	0.480*	0.202*	0.414*	0.129*	0.130	0.145*	0.154*
	(0.029)	(0.030)	(0.065)	(0.097)	(0.065)	(0.080)	(0.061)	(0.060)
other non-communicable diseases	-0.205*	-0.025	0.367*	0.243*	0.128*	0.213*	0.112*	0.142*
	(0.083)	(0.098)	(0.040)	(0.090)	(0.036)	(0.038)	(0.036)	(0.028)
well care	-0.432*	-0.176*	-0.089*	-1.165*	0.050	-1.088*	0.162*	-0.015
	(0.021)	(0.020)	(0.027)	(0.129)	(0.031)	(0.103)	(0.035)	(0.117)
Constant	4.875*	4.851*	3.128*	2.656*	3.247*	3.156*	3.276*	3.689*
	(0.009)	(0.008)	(0.021)	(0.022)	(0.027)	(0.021)	(0.039)	(0.028)
Adjusted R^2	0.12	0.14	0.07	0.06	0.04	0.07	0.04	0.06
Observations	66567	71059	127532	120801	106368	102632	61884	52945

b coefficients; se in parentheses; f: female, m:male

* $p < 0.05$

The adj. R2 was defined as 1-[(1-R2)*(n-1)/dof]; R2 was defined as the correlation between observed and predicted values on the original scale

3 Regression output: Hospital outpatient

TABLE 3. REGRESSION COEFFICIENTS AND STANDARD ERRORS FROM POISSON PSEUDO-MAXIMUM LIKELIHOOD MODELS

	0-19 y. (f)	0-19 y. (m)	20-44 y. (f)	20-44 y. (m)	45-64 y. (f)	45-64 y. (m)	65+ y. (f)	65+ y. (m)
HIV/AIDS	0.962	0.048	0.923*	0.980*	0.622*	0.830*	0.649*	0.607*
	(0.497)	(0.247)	(0.144)	(0.132)	(0.153)	(0.096)	(0.247)	(0.144)
other communicable diseases	0.631*	0.641*	0.503*	0.695*	0.438*	0.634*	0.400*	0.542*
	(0.029)	(0.028)	(0.027)	(0.036)	(0.029)	(0.033)	(0.029)	(0.029)
hepatitis			1.320*	0.550	0.503*	1.069*	-0.108	0.771*
			(0.197)	(0.452)	(0.194)	(0.207)	(0.307)	(0.245)
maternal and neonatal disorders	1.529*	0.695*	0.025		0.195			
	(0.265)	(0.349)	(0.095)		(0.457)			
nutritional deficiencies	0.173*	0.492*	0.170*	0.244	0.145*	0.209*	0.265*	0.226*
	(0.053)	(0.059)	(0.029)	(0.126)	(0.047)	(0.086)	(0.046)	(0.053)
colon and rectum cancers	0.972*		0.517*	0.328	0.345*	1.316*	0.673*	0.926*
	(0.302)		(0.231)	(0.491)	(0.110)	(0.129)	(0.086)	(0.088)
trachea, bronchus, and lung cancers	2.030*	0.381	0.894*	1.216*	1.554*	0.920*	1.097*	0.984*
	(0.369)	(0.484)	(0.240)	(0.307)	(0.134)	(0.160)	(0.101)	(0.087)
breast cancer			1.729*		1.935*		1.398*	
			(0.154)		(0.077)		(0.065)	
prostate cancer		1.855*		1.208*		1.028*		1.101*
		(0.369)		(0.414)		(0.165)		(0.064)
other neoplasms	1.415*	1.752*	1.041*	1.689*	1.278*	1.478*	1.220*	0.963*
	(0.252)	(0.352)	(0.137)	(0.130)	(0.077)	(0.076)	(0.063)	(0.053)
ischemic heart disease	-0.044	0.072	0.297*	0.596*	0.528*	0.949*	0.538*	0.606*
	(0.228)	(0.220)	(0.091)	(0.090)	(0.062)	(0.058)	(0.049)	(0.044)
stroke	1.114*	-2.056*	1.640*	0.810*	0.843*	0.346*	0.440*	0.455*
	(0.018)	(0.018)	(0.233)	(0.188)	(0.175)	(0.170)	(0.204)	(0.073)
hypertensive heart disease			0.543	0.886*	0.696*	0.235	0.388*	0.306*
			(0.342)	(0.337)	(0.233)	(0.303)	(0.160)	(0.152)
atrial fibrillation and flutter	0.396	0.597	0.530	1.014*	0.726*	1.082*	0.414*	0.370*
	(0.643)	(0.883)	(0.343)	(0.279)	(0.202)	(0.139)	(0.086)	(0.060)
other cardiovascular and circulatory diseases	0.687*	0.791*	0.309*	0.523*	0.287*	0.348*	0.118*	0.190*
	(0.120)	(0.089)	(0.050)	(0.059)	(0.035)	(0.035)	(0.030)	(0.031)

Table 3. Regression coefficients and standard errors from Poisson pseudo-maximum likelihood models

	0-19 y. (f)	0-19 y. (m)	20-44 y. (f)	20-44 y. (m)	45-64 y. (f)	45-64 y. (m)	65+ y. (f)	65+ y. (m)
copd	0.371	0.254	0.219	0.193	0.184	0.365*	0.145	0.066
	(0.293)	(0.237)	(0.159)	(0.200)	(0.104)	(0.089)	(0.074)	(0.061)
asthma	0.621*	0.634*	0.118*	0.191*	0.035	0.010	0.037	-0.004
	(0.050)	(0.052)	(0.050)	(0.077)	(0.059)	(0.063)	(0.055)	(0.057)
other chronic respiratory diseases	0.459*	0.322*	0.380*	0.324*	0.049	0.211*	0.051	0.014
	(0.153)	(0.162)	(0.082)	(0.109)	(0.088)	(0.094)	(0.075)	(0.069)
cirrhosis and other chronic liver diseases			0.500	1.402*	0.739	1.082*	0.858*	0.513
			(0.364)	(0.184)	(0.391)	(0.179)	(0.319)	(0.362)
other digestive diseases	0.981*	0.975*	0.958*	0.787*	0.644*	0.442*	0.469*	0.380*
	(0.046)	(0.041)	(0.034)	(0.043)	(0.031)	(0.034)	(0.031)	(0.031)
alzheimer's disease and other dementias	-0.087	0.888*	0.214	0.501*	-0.029	0.035	0.101*	-0.034
	(0.393)	(0.404)	(0.146)	(0.167)	(0.075)	(0.112)	(0.051)	(0.057)
parkinson's disease	-0.588	0.821*	0.837*	0.135	0.172	0.473*	0.159*	0.209*
	(0.509)	(0.298)	(0.123)	(0.254)	(0.104)	(0.190)	(0.062)	(0.071)
epilepsy	1.472*	1.133*	1.068*	1.117*	0.796*	0.620*	0.435*	0.396*
	(0.128)	(0.143)	(0.080)	(0.088)	(0.064)	(0.073)	(0.052)	(0.048)
multiple sclerosis	2.020*	3.409*	1.399*	1.609*	0.927*	0.877*	0.381	0.278
	(0.195)	(0.037)	(0.125)	(0.206)	(0.119)	(0.204)	(0.319)	(0.545)
other neurological diseases	0.945*	1.020*	0.604*	0.822*	0.423*	0.566*	0.354*	0.388*
	(0.066)	(0.056)	(0.039)	(0.051)	(0.038)	(0.043)	(0.046)	(0.042)
schizophrenia	0.803*	-0.620	-0.285	-0.038	0.007	-0.212	-0.198	0.086
	(0.315)	(0.605)	(0.216)	(0.198)	(0.179)	(0.198)	(0.143)	(0.278)
depression	0.436*	0.614*	0.332*	0.587*	0.364*	0.341*	0.023	0.065
	(0.113)	(0.152)	(0.038)	(0.068)	(0.042)	(0.045)	(0.036)	(0.046)
adhd	0.443*	0.193*	0.452*	0.423*	-0.005	0.228	0.332	0.043
	(0.106)	(0.076)	(0.136)	(0.140)	(0.121)	(0.179)	(0.253)	(0.204)
other mental disorders	0.692*	0.636*	0.351*	0.481*	0.423*	0.355*	0.168*	0.080
	(0.053)	(0.055)	(0.045)	(0.054)	(0.044)	(0.051)	(0.051)	(0.049)
alcohol and drug use disorders	1.385*	0.245	-0.609	0.038	0.119	0.237*	0.388*	0.380
	(0.384)	(0.353)	(0.476)	(0.141)	(0.173)	(0.120)	(0.161)	(0.197)
diabetes	1.252*	1.899*	0.397*	0.435*	0.177*	0.165*	0.081*	0.147*
	(0.255)	(0.107)	(0.092)	(0.116)	(0.056)	(0.047)	(0.039)	(0.035)
chronic kidney disease		-1.484*	0.679*	0.456	0.394*	0.379*	0.513*	0.497*
		(0.456)	(0.260)	(0.318)	(0.193)	(0.168)	(0.137)	(0.101)

Table 3. Regression coefficients and standard errors from Poisson pseudo-maximum likelihood models

	0-19 y. (f)	0-19 y. (m)	20-44 y. (f)	20-44 y. (m)	45-64 y. (f)	45-64 y. (m)	65+ y. (f)	65+ y. (m)
skin and subcutaneous diseases	0.136*	0.303*	0.161*	0.207*	0.169*	0.076*	0.123*	0.033
	(0.032)	(0.031)	(0.030)	(0.040)	(0.033)	(0.039)	(0.032)	(0.030)
sense organ diseases	0.414*	0.370*	0.298*	0.390*	0.141*	0.162*	0.333*	0.255*
	(0.029)	(0.028)	(0.030)	(0.040)	(0.030)	(0.033)	(0.030)	(0.028)
rheumatoid arthritis	1.735*	1.172*	0.603*	0.933*	0.585*	0.540*	0.168*	0.222*
	(0.208)	(0.456)	(0.096)	(0.102)	(0.074)	(0.087)	(0.074)	(0.097)
osteoarthritis	-0.031	-0.279	0.051	0.435*	0.159*	0.250*	-0.015	-0.030
	(0.232)	(0.373)	(0.150)	(0.148)	(0.048)	(0.081)	(0.041)	(0.057)
low back pain	0.789*	0.218	0.948*	0.999*	0.808*	0.756*	0.624*	0.423*
	(0.264)	(0.256)	(0.091)	(0.097)	(0.076)	(0.078)	(0.065)	(0.078)
osteoporosis	0.602	1.943*	0.747*	0.506*	0.420*	0.426*	0.178*	0.300*
	(0.406)	(0.275)	(0.114)	(0.152)	(0.056)	(0.096)	(0.036)	(0.055)
other musculoskeletal disorders	0.465*	0.333*	0.463*	0.438*	0.366*	0.394*	0.191*	0.154*
	(0.036)	(0.036)	(0.029)	(0.038)	(0.032)	(0.032)	(0.033)	(0.029)
oral disorders	0.177*	0.303*	0.088	0.113	0.151*	0.112	0.341*	0.332*
	(0.067)	(0.079)	(0.068)	(0.081)	(0.063)	(0.088)	(0.081)	(0.066)
other non-communicable diseases	0.188*	0.441*	0.272*	0.560*	0.227*	0.433*	0.170*	0.234*
	(0.090)	(0.132)	(0.037)	(0.065)	(0.035)	(0.035)	(0.030)	(0.028)
well care	-0.149*	-0.186*	-0.012	-1.701*	-0.098*	-0.888*	-0.004	-0.565*
	(0.034)	(0.041)	(0.024)	(0.112)	(0.027)	(0.097)	(0.042)	(0.107)
Constant	4.076*	4.135*	4.196*	3.960*	4.620*	4.659*	5.036*	5.399*
	(0.018)	(0.018)	(0.021)	(0.017)	(0.024)	(0.020)	(0.033)	(0.030)
Adjusted R^2	0.21	0.09	0.15	0.18	0.17	0.12	0.11	0.13
Observations	66567	71059	127532	120801	106368	102632	61884	52945

b coefficients; se in parentheses; f: female, m:male

* $p < 0.05$

The adj. R2 was defined as 1-[(1-R2)*(n-1)/dof]; R2 was defined as the correlation between observed and predicted values on the original scale

4 Regression output: Drugs outpatient

TABLE 4. REGRESSION COEFFICIENTS AND STANDARD ERRORS FROM POISSON PSEUDO-MAXIMUM LIKELIHOOD MODELS

	0-19 y. (f)	0-19 y. (m)	20-44 y. (f)	20-44 y. (m)	45-64 y. (f)	45-64 y. (m)	65+ y. (f)	65+ y. (m)
HIV/AIDS	0.378	-3.256*	0.224	1.340*	0.723*	0.376*	0.708	0.549*
	(0.552)	(0.481)	(0.471)	(0.364)	(0.140)	(0.164)	(0.442)	(0.185)
hepatitis			0.465	0.790	0.351	0.134	0.590	0.057
			(0.630)	(0.525)	(0.234)	(0.284)	(0.646)	(0.372)
maternal and neonatal disorders	0.742	0.015	-0.131		-0.967*			
	(0.700)	(0.467)	(0.166)		(0.374)			
nutritional deficiencies	0.800*	0.731*	0.051	0.846	0.363*	0.293*	0.353*	0.302*
	(0.156)	(0.238)	(0.075)	(0.520)	(0.139)	(0.100)	(0.042)	(0.056)
colon and rectum cancers	1.929*		0.692	1.002	0.384*	0.957*	0.245*	0.713*
	(0.373)		(0.372)	(0.872)	(0.148)	(0.258)	(0.084)	(0.099)
trachea, bronchus, and lung cancers	0.209	2.814	0.066	1.053	0.641*	0.932*	0.721*	0.929*
	(0.379)	(1.475)	(0.604)	(0.886)	(0.187)	(0.379)	(0.150)	(0.169)
breast cancer			0.859*		0.637*		0.460*	
			(0.254)		(0.101)		(0.100)	
prostate cancer		0.805		-0.021		0.671*		0.153*
		(0.507)		(0.922)		(0.277)		(0.060)
other neoplasms	0.982*	1.344*	0.495*	1.320*	0.643*	0.905*	0.871*	0.533*
	(0.435)	(0.284)	(0.152)	(0.256)	(0.114)	(0.136)	(0.172)	(0.071)
ischemic heart disease	0.134	0.529	0.269	-0.096	0.549*	0.934*	0.331*	0.378*
	(0.167)	(0.352)	(0.335)	(0.264)	(0.139)	(0.224)	(0.062)	(0.052)
stroke	1.080*	0.100	0.261	-0.020	0.432*	0.057	0.194*	0.174*
	(0.050)	(0.056)	(0.346)	(0.323)	(0.186)	(0.253)	(0.067)	(0.054)
hypertensive heart disease			-0.426	-0.216	0.546	0.533	0.719*	0.474
			(0.652)	(0.724)	(0.358)	(0.315)	(0.203)	(0.244)
atrial fibrillation and flutter	-0.636	0.209	0.693	-0.181	0.177	0.708*	0.629*	0.596*
	(0.443)	(0.501)	(0.505)	(0.376)	(0.127)	(0.156)	(0.063)	(0.072)
other cardiovascular and circulatory diseases	0.590	0.380*	0.349*	0.144	0.405*	0.476*	0.271*	0.272*
	(0.368)	(0.140)	(0.098)	(0.204)	(0.050)	(0.074)	(0.055)	(0.042)
copd	0.981	0.343	-0.243	1.910*	0.387*	0.248	0.131*	0.187*
	(0.685)	(0.225)	(0.205)	(0.645)	(0.150)	(0.145)	(0.047)	(0.091)

Table 4. Regression coefficients and standard errors from Poisson pseudo-maximum likelihood models

	0-19 y. (f)	0-19 y. (m)	20-44 y. (f)	20-44 y. (m)	45-64 y. (f)	45-64 y. (m)	65+ y. (f)	65+ y. (m)
asthma	0.727*	0.795*	0.141	0.490*	0.087	0.278*	0.159*	0.096
	(0.097)	(0.092)	(0.089)	(0.172)	(0.067)	(0.084)	(0.037)	(0.069)
other chronic respiratory diseases	0.739*	0.591*	0.252	0.012	0.092	0.142	0.321*	0.228*
	(0.374)	(0.214)	(0.149)	(0.152)	(0.106)	(0.135)	(0.108)	(0.077)
cirrhosis and other chronic liver diseases			0.788	0.506	0.671*	0.337	0.246	1.274*
			(0.500)	(0.583)	(0.272)	(0.272)	(0.140)	(0.274)
other digestive diseases	0.741*	0.613*	0.644*	0.429*	0.437*	0.594*	0.278*	0.280*
	(0.120)	(0.118)	(0.112)	(0.115)	(0.050)	(0.092)	(0.038)	(0.037)
alzheimer's disease and other dementias	0.251	0.431	0.426*	0.519	0.301*	0.294*	0.114*	0.170*
	(0.235)	(0.405)	(0.169)	(0.300)	(0.065)	(0.082)	(0.041)	(0.055)
parkinson's disease	-0.664	1.064*	0.928*	1.023*	0.536*	0.574*	0.396*	0.452*
	(0.814)	(0.333)	(0.241)	(0.312)	(0.119)	(0.173)	(0.052)	(0.081)
epilepsy	0.783*	0.240	1.093*	1.162*	0.791*	0.795*	0.416*	0.491*
	(0.334)	(0.154)	(0.137)	(0.249)	(0.083)	(0.104)	(0.041)	(0.062)
multiple sclerosis	0.642*	0.782*	2.507*	2.792*	2.395*	2.062*	2.655*	1.033*
	(0.120)	(0.122)	(0.188)	(0.331)	(0.132)	(0.221)	(0.193)	(0.505)
other neurological diseases	0.257*	0.398*	0.667*	0.630*	0.555*	0.521*	0.272*	0.290*
	(0.096)	(0.116)	(0.118)	(0.130)	(0.058)	(0.067)	(0.041)	(0.038)
schizophrenia	0.803*	0.135	0.081	0.088	0.405*	0.888*	0.596*	0.976*
	(0.387)	(0.383)	(0.156)	(0.201)	(0.205)	(0.212)	(0.251)	(0.340)
depression	0.354*	0.240	0.497*	0.433*	0.552*	0.585*	0.539*	0.472*
	(0.173)	(0.146)	(0.067)	(0.166)	(0.065)	(0.070)	(0.075)	(0.052)
adhd	1.112*	0.865*	0.572*	1.001*	0.635*	0.459*	0.191	0.165
	(0.420)	(0.297)	(0.147)	(0.367)	(0.271)	(0.150)	(0.213)	(0.274)
other mental disorders	0.433*	0.220*	0.554*	0.753*	0.427*	0.481*	0.537*	0.494*
	(0.135)	(0.082)	(0.159)	(0.143)	(0.059)	(0.064)	(0.040)	(0.047)
alcohol and drug use disorders	0.410	0.451	-0.167	1.410*	0.377*	0.683*	0.387*	0.116
	(0.420)	(0.553)	(0.277)	(0.441)	(0.107)	(0.172)	(0.148)	(0.110)
diabetes	1.668*	1.963*	0.695*	0.983*	0.208	0.093	0.087*	0.061
	(0.549)	(0.643)	(0.280)	(0.439)	(0.177)	(0.070)	(0.033)	(0.038)
chronic kidney disease		0.470	1.006*	-0.208	0.470	0.532*	0.707*	0.919*
		(0.657)	(0.406)	(0.644)	(0.257)	(0.236)	(0.123)	(0.129)
skin and subcutaneous diseases	0.509*	0.585*	0.397*	0.665*	0.268*	0.446*	0.202*	0.132*
	(0.070)	(0.071)	(0.080)	(0.134)	(0.053)	(0.090)	(0.046)	(0.033)

Table 4. Regression coefficients and standard errors from Poisson pseudo-maximum likelihood models

	0-19 y. (f)	0-19 y. (m)	20-44 y. (f)	20-44 y. (m)	45-64 y. (f)	45-64 y. (m)	65+ y. (f)	65+ y. (m)
sense organ diseases	0.520*	0.476*	0.392*	0.219	0.197*	0.062	0.042	0.112*
	(0.086)	(0.079)	(0.108)	(0.119)	(0.050)	(0.058)	(0.044)	(0.037)
rheumatoid arthritis	1.478*	0.914	1.612*	1.765*	1.056*	1.062*	0.608*	0.596*
	(0.400)	(0.986)	(0.199)	(0.213)	(0.079)	(0.131)	(0.091)	(0.090)
osteoarthritis	0.557*	0.092	0.020	-0.058	0.299*	0.338*	0.096*	0.142*
	(0.203)	(0.159)	(0.184)	(0.246)	(0.053)	(0.167)	(0.041)	(0.049)
low back pain	0.362	-0.150	0.684*	1.018	0.392*	0.281*	0.323*	0.215*
	(0.221)	(0.214)	(0.157)	(0.552)	(0.062)	(0.126)	(0.067)	(0.076)
osteoporosis	1.859*	1.599*	0.760*	1.043*	0.494*	0.604*	0.505*	0.589*
	(0.920)	(0.324)	(0.205)	(0.364)	(0.050)	(0.131)	(0.056)	(0.064)
other musculoskeletal disorders	0.351*	0.388*	0.595*	0.464*	0.500*	0.339*	0.351*	0.314*
	(0.102)	(0.109)	(0.095)	(0.217)	(0.060)	(0.068)	(0.026)	(0.039)
oral disorders	0.134	0.086	-0.078	0.055	0.177*	0.331*	0.289*	0.338*
	(0.102)	(0.084)	(0.101)	(0.161)	(0.060)	(0.106)	(0.057)	(0.061)
other non-communicable diseases	0.599*	0.809	0.583*	0.779*	0.357*	0.406*	0.201*	0.215*
	(0.294)	(0.432)	(0.090)	(0.291)	(0.056)	(0.080)	(0.046)	(0.039)
well care	-0.131*	-0.280*	0.101	-0.464*	-0.063	-0.275	-0.176*	-0.291*
	(0.056)	(0.056)	(0.074)	(0.148)	(0.043)	(0.188)	(0.033)	(0.098)
Constant	3.432*	3.506*	4.145*	4.063*	4.687*	4.495*	5.288*	5.326*
	(0.050)	(0.056)	(0.069)	(0.138)	(0.039)	(0.048)	(0.037)	(0.033)
-								
other communicable diseases								
Adjusted R^2	0.01	0.01	0.01	0.01	0.02	0.02	0.04	0.05
Observations	66567	71059	127532	120801	106368	102632	61884	52945

b coefficients; se in parentheses; f: female, m:male

* $p < 0.05$

The adj. R2 was defined as 1-[(1-R2)*(n-1)/dof]; R2 was defined as the correlation between observed and predicted values on the original scale

5 Regression output: Laboratory

Table 5. Regression coefficients and standard errors from Poisson pseudo-maximum likelihood models

	0-19 y. (f)	0-19 y. (m)	20-44 y. (f)	20-44 y. (m)	45-64 y. (f)	45-64 y. (m)	65+ y. (f)	65+ y. (m)
HIV/AIDS	0.763	1.084*	1.136*	1.835*	1.329*	1.564*	1.645*	1.405*
	(0.431)	(0.368)	(0.111)	(0.078)	(0.130)	(0.078)	(0.389)	(0.164)
other communicable diseases	0.812*	0.736*	0.534*	0.789*	0.474*	0.587*	0.411*	0.493*
	(0.022)	(0.029)	(0.010)	(0.021)	(0.012)	(0.016)	(0.012)	(0.015)
hepatitis			1.548*	0.757*	0.933*	1.309*	1.041*	1.565*
			(0.120)	(0.259)	(0.222)	(0.100)	(0.191)	(0.161)
maternal and neonatal disorders	0.401	-0.203	0.237*		0.509			
	(0.329)	(0.282)	(0.040)		(0.398)			
nutritional deficiencies	1.095*	1.324*	0.513*	0.562*	0.513*	0.356*	0.413*	0.338*
	(0.028)	(0.043)	(0.011)	(0.088)	(0.017)	(0.051)	(0.020)	(0.046)
colon and rectum cancers	0.028		0.564*	-0.236	0.494*	0.855*	0.607*	0.520*
	(0.422)		(0.165)	(0.425)	(0.073)	(0.091)	(0.053)	(0.063)
trachea, bronchus, and lung cancers	1.822*	0.521	0.112	1.019*	0.901*	0.632*	0.809*	0.562*
	(0.334)	(0.334)	(0.175)	(0.460)	(0.088)	(0.107)	(0.077)	(0.078)
breast cancer			0.555*		0.665*		0.554*	
			(0.105)		(0.046)		(0.035)	
prostate cancer		1.835*		1.387*		0.541*		0.548*
		(0.544)		(0.368)		(0.112)		(0.037)
other neoplasms	0.854*	1.683*	0.515*	0.952*	0.726*	0.823*	0.697*	0.652*
	(0.181)	(0.441)	(0.059)	(0.142)	(0.055)	(0.074)	(0.043)	(0.050)
ischemic heart disease	0.193	0.621*	0.193*	0.371*	0.391*	0.508*	0.377*	0.400*
	(0.133)	(0.183)	(0.041)	(0.070)	(0.028)	(0.037)	(0.021)	(0.025)
stroke	-0.132*		0.326	-0.353	0.585*	0.143	0.195*	0.329*
	(0.015)		(0.337)	(0.240)	(0.136)	(0.093)	(0.057)	(0.064)
hypertensive heart disease			0.249	0.839*	0.289*	0.829*	0.547*	0.410*
			(0.462)	(0.293)	(0.136)	(0.211)	(0.084)	(0.100)
atrial fibrillation and flutter	0.935*	0.121	0.331*	0.266	0.496*	0.491*	0.337*	0.303*
	(0.380)	(0.397)	(0.156)	(0.371)	(0.114)	(0.086)	(0.043)	(0.037)
other cardiovascular and circulatory diseases	0.347*	0.834*	0.172*	0.371*	0.190*	0.266*	0.123*	0.197*
	(0.116)	(0.103)	(0.020)	(0.039)	(0.015)	(0.019)	(0.014)	(0.018)

Table 5. Regression coefficients and standard errors from Poisson pseudo-maximum likelihood models

	0-19 y. (f)	0-19 y. (m)	20-44 y. (f)	20-44 y. (m)	45-64 y. (f)	45-64 y. (m)	65+ y. (f)	65+ y. (m)
copd	0.443*	-0.094	0.015	0.108	-0.023	0.050	0.095*	-0.023
	(0.217)	(0.240)	(0.129)	(0.171)	(0.053)	(0.067)	(0.032)	(0.038)
asthma	0.403*	0.573*	0.125*	0.112	0.047	0.098*	0.057*	0.007
	(0.049)	(0.083)	(0.031)	(0.067)	(0.029)	(0.036)	(0.022)	(0.028)
other chronic respiratory diseases	0.578*	0.779*	0.401*	0.225*	0.168*	0.165*	0.190*	0.116*
	(0.163)	(0.187)	(0.051)	(0.084)	(0.053)	(0.063)	(0.043)	(0.045)
cirrhosis and other chronic liver diseases			0.773*	1.733*	0.960*	1.387*	0.957*	1.080*
			(0.294)	(0.306)	(0.161)	(0.191)	(0.222)	(0.113)
other digestive diseases	0.577*	0.528*	0.574*	0.750*	0.504*	0.558*	0.401*	0.391*
	(0.042)	(0.055)	(0.015)	(0.030)	(0.015)	(0.019)	(0.013)	(0.019)
alzheimer's disease and other dementias	0.164	0.446*	0.127*	0.265*	0.174*	0.071	0.084*	0.104*
	(0.258)	(0.182)	(0.060)	(0.112)	(0.035)	(0.082)	(0.020)	(0.033)
parkinson's disease	0.591*	0.996*	0.327*	0.112	0.077	0.206*	0.048	0.016
	(0.287)	(0.209)	(0.102)	(0.170)	(0.053)	(0.075)	(0.028)	(0.039)
epilepsy	0.823*	0.695*	0.481*	0.643*	0.378*	0.428*	0.217*	0.314*
	(0.141)	(0.197)	(0.044)	(0.076)	(0.039)	(0.054)	(0.029)	(0.055)
multiple sclerosis	0.925*	-0.395*	0.638*	0.795*	0.604*	0.681*	0.510*	0.562*
	(0.413)	(0.039)	(0.077)	(0.209)	(0.080)	(0.167)	(0.202)	(0.242)
other neurological diseases	0.368*	0.491*	0.212*	0.340*	0.192*	0.310*	0.203*	0.228*
	(0.063)	(0.067)	(0.016)	(0.036)	(0.017)	(0.026)	(0.018)	(0.029)
schizophrenia	0.388*	1.461*	0.213*	0.628*	0.233*	0.272*	0.225*	0.146
	(0.183)	(0.397)	(0.089)	(0.103)	(0.066)	(0.075)	(0.072)	(0.103)
depression	0.562*	0.782*	0.371*	0.540*	0.255*	0.301*	0.070*	0.093*
	(0.077)	(0.114)	(0.018)	(0.033)	(0.015)	(0.025)	(0.015)	(0.031)
adhd	0.486*	0.469*	0.322*	0.248*	0.107	-0.016	0.188	0.270
	(0.103)	(0.061)	(0.049)	(0.067)	(0.078)	(0.088)	(0.136)	(0.194)
other mental disorders	0.653*	0.512*	0.377*	0.487*	0.264*	0.232*	0.079*	0.016
	(0.040)	(0.053)	(0.017)	(0.035)	(0.020)	(0.031)	(0.024)	(0.052)
alcohol and drug use disorders	0.934*	0.780*	-0.189	0.335*	0.028	0.262*	0.009	-0.114
	(0.238)	(0.289)	(0.183)	(0.093)	(0.073)	(0.075)	(0.097)	(0.118)
diabetes	1.011*	1.514*	0.331*	0.310*	0.148*	0.232*	0.199*	0.194*
	(0.152)	(0.118)	(0.042)	(0.078)	(0.028)	(0.027)	(0.017)	(0.028)
chronic kidney disease		-1.471*	0.485	-0.179	0.147	0.051	0.637*	0.777*
		(0.403)	(0.263)	(0.331)	(0.163)	(0.171)	(0.084)	(0.129)

Table 5. Regression coefficients and standard errors from Poisson pseudo-maximum likelihood models

	0-19 y. (f)	0-19 y. (m)	20-44 y. (f)	20-44 y. (m)	45-64 y. (f)	45-64 y. (m)	65+ y. (f)	65+ y. (m)
skin and subcutaneous diseases	0.338*	0.367*	0.267*	0.431*	0.224*	0.287*	0.173*	0.153*
	(0.024)	(0.032)	(0.011)	(0.024)	(0.014)	(0.021)	(0.013)	(0.019)
sense organ diseases	0.187*	0.169*	0.174*	0.260*	0.134*	0.157*	0.152*	0.095*
	(0.023)	(0.030)	(0.013)	(0.030)	(0.012)	(0.018)	(0.013)	(0.021)
rheumatoid arthritis	1.786*	1.796*	0.706*	0.994*	0.720*	0.675*	0.476*	0.572*
	(0.171)	(0.268)	(0.058)	(0.104)	(0.050)	(0.078)	(0.047)	(0.065)
osteoarthritis	0.724*	0.139	0.167*	0.440*	0.209*	0.281*	0.165*	0.155*
	(0.153)	(0.404)	(0.068)	(0.096)	(0.027)	(0.043)	(0.018)	(0.028)
low back pain	0.280	0.128	0.154*	0.154	0.208*	0.101*	0.239*	0.010
	(0.173)	(0.230)	(0.060)	(0.100)	(0.042)	(0.050)	(0.035)	(0.043)
osteoporosis	0.563*	1.903*	0.659*	0.746*	0.427*	0.565*	0.250*	0.397*
	(0.253)	(0.309)	(0.053)	(0.119)	(0.025)	(0.066)	(0.018)	(0.038)
other musculoskeletal disorders	0.437*	0.342*	0.312*	0.395*	0.323*	0.300*	0.237*	0.202*
	(0.025)	(0.036)	(0.011)	(0.023)	(0.014)	(0.017)	(0.014)	(0.015)
oral disorders	0.227*	0.386*	0.117*	0.264*	0.144*	0.167*	0.235*	0.179*
	(0.048)	(0.100)	(0.027)	(0.066)	(0.035)	(0.060)	(0.041)	(0.091)
other non-communicable diseases	0.732*	0.935*	0.614*	0.698*	0.398*	0.402*	0.255*	0.211*
	(0.071)	(0.138)	(0.014)	(0.048)	(0.014)	(0.020)	(0.012)	(0.023)
well care	0.243*	-0.356*	0.161*	-0.566*	0.088*	-0.461*	0.089*	-0.351*
	(0.024)	(0.045)	(0.010)	(0.038)	(0.011)	(0.062)	(0.014)	(0.062)
Constant	3.576*	3.497*	4.533*	3.961*	4.598*	4.426*	4.792*	4.940*
	(0.015)	(0.018)	(0.008)	(0.012)	(0.010)	(0.011)	(0.014)	(0.014)
Adjusted R^2	0.18	0.10	0.34	0.22	0.26	0.28	0.26	0.20
Observations	66567	71059	127532	120801	106368	102632	61884	52945

b coefficients; se in parentheses; f: female, m:male

* $p < 0.05$

The adj. R2 was defined as 1-[(1-R2)*(n-1)/dof]; R2 was defined as the correlation between observed and predicted values on the original scale

6 Regression output: Radiology

Table 6. Regression coefficients and standard errors from Poisson pseudo-maximum likelihood models

	0-19 y. (f)	0-19 y. (m)	20-44 y. (f)	20-44 y. (m)	45-64 y. (f)	45-64 y. (m)	65+ y. (f)	65+ y. (m)
HIV/AIDS	0.212	0.214	-0.099	0.372*	0.276*	0.111	0.411*	0.532*
	(0.573)	(0.453)	(0.177)	(0.115)	(0.116)	(0.096)	(0.172)	(0.120)
other communicable diseases	0.380*	0.237*	0.349*	0.463*	0.306*	0.497*	0.304*	0.461*
	(0.038)	(0.042)	(0.017)	(0.027)	(0.014)	(0.019)	(0.017)	(0.019)
hepatitis			0.928*	0.535	0.600*	0.504*	-0.131	0.210
			(0.185)	(0.317)	(0.202)	(0.161)	(0.240)	(0.216)
maternal and neonatal disorders	1.368*	1.072*	-0.014		-0.672			
	(0.214)	(0.242)	(0.054)		(0.488)			
nutritional deficiencies	0.328*	0.494*	0.136*	0.262*	0.136*	0.193*	0.194*	0.183*
	(0.053)	(0.098)	(0.019)	(0.099)	(0.022)	(0.060)	(0.029)	(0.041)
colon and rectum cancers	1.716*		0.753*	0.675	0.474*	1.074*	0.754*	0.864*
	(0.608)		(0.145)	(0.349)	(0.076)	(0.089)	(0.055)	(0.062)
trachea, bronchus, and lung cancers	1.225	1.616*	0.519*	0.648*	1.038*	0.634*	0.901*	0.896*
	(0.712)	(0.184)	(0.174)	(0.228)	(0.091)	(0.112)	(0.069)	(0.076)
breast cancer			0.981*		0.884*		0.778*	
			(0.099)		(0.044)		(0.041)	
prostate cancer		0.636		0.585		0.836*		0.864*
		(0.359)		(0.697)		(0.090)		(0.039)
other neoplasms	1.065*	1.426*	0.618*	1.150*	0.658*	0.938*	0.688*	0.502*
	(0.422)	(0.394)	(0.077)	(0.102)	(0.047)	(0.051)	(0.037)	(0.038)
ischemic heart disease	0.371*	0.561*	0.332*	0.560*	0.383*	0.469*	0.331*	0.342*
	(0.165)	(0.191)	(0.051)	(0.067)	(0.033)	(0.038)	(0.028)	(0.031)
stroke			1.161*	1.497*	0.692*	0.530*	0.118	0.207*
			(0.338)	(0.237)	(0.150)	(0.118)	(0.063)	(0.061)
hypertensive heart disease			-0.270	0.015	0.206	-0.012	0.061	0.336*
			(0.679)	(0.515)	(0.178)	(0.172)	(0.131)	(0.122)
atrial fibrillation and flutter	1.915*		0.035	0.042	-0.009	0.285*	0.060	0.038
	(0.725)		(0.207)	(0.217)	(0.127)	(0.093)	(0.056)	(0.046)
other cardiovascular and circulatory diseases	0.723*	0.618*	0.261*	0.411*	0.172*	0.215*	0.045*	0.112*
	(0.132)	(0.149)	(0.029)	(0.055)	(0.017)	(0.021)	(0.017)	(0.020)

Table 6. Regression coefficients and standard errors from Poisson pseudo-maximum likelihood models

	0-19 y. (f)	0-19 y. (m)	20-44 y. (f)	20-44 y. (m)	45-64 y. (f)	45-64 y. (m)	65+ y. (f)	65+ y. (m)
copd	-0.029	-0.049	-0.185	-0.332	0.060	0.208*	0.059	0.013
	(0.365)	(0.327)	(0.147)	(0.210)	(0.063)	(0.066)	(0.044)	(0.044)
asthma	0.093	0.174*	0.025	0.181*	0.038	0.013	0.082*	0.023
	(0.074)	(0.084)	(0.046)	(0.063)	(0.033)	(0.037)	(0.031)	(0.035)
other chronic respiratory diseases	0.103	0.770*	0.303*	0.368*	0.252*	0.143*	0.332*	0.211*
	(0.191)	(0.235)	(0.069)	(0.085)	(0.053)	(0.049)	(0.049)	(0.045)
cirrhosis and other chronic liver diseases			0.040	1.472*	0.801*	0.632*	0.701*	0.680*
			(0.400)	(0.377)	(0.215)	(0.288)	(0.272)	(0.164)
other digestive diseases	0.299*	0.297*	0.586*	0.714*	0.467*	0.525*	0.521*	0.405*
	(0.065)	(0.070)	(0.021)	(0.032)	(0.016)	(0.020)	(0.018)	(0.020)
alzheimer's disease and other dementias	0.304	0.955*	0.494*	0.484*	0.305*	0.341*	0.177*	0.129*
	(0.342)	(0.483)	(0.093)	(0.127)	(0.040)	(0.067)	(0.029)	(0.041)
parkinson's disease	-1.338*	-1.120	0.679*	0.188	0.388*	0.399*	0.212*	0.114*
	(0.529)	(0.838)	(0.112)	(0.217)	(0.062)	(0.088)	(0.039)	(0.048)
epilepsy	1.200*	1.400*	0.906*	0.814*	0.545*	0.551*	0.320*	0.328*
	(0.199)	(0.210)	(0.052)	(0.066)	(0.033)	(0.041)	(0.030)	(0.035)
multiple sclerosis	4.048*	2.431*	1.955*	2.183*	1.348*	1.218*	0.895*	0.735
	(0.152)	(0.037)	(0.082)	(0.134)	(0.088)	(0.179)	(0.254)	(0.393)
other neurological diseases	0.967*	0.929*	0.708*	0.709*	0.454*	0.543*	0.442*	0.432*
	(0.076)	(0.089)	(0.022)	(0.034)	(0.018)	(0.024)	(0.022)	(0.026)
schizophrenia	-2.950*	-1.670*	-0.469*	-0.322	-0.293*	-0.288*	-0.426*	-0.385
	(0.873)	(0.536)	(0.132)	(0.173)	(0.094)	(0.129)	(0.128)	(0.222)
depression	0.446*	0.543*	0.289*	0.307*	0.186*	0.206*	-0.008	0.056
	(0.105)	(0.189)	(0.028)	(0.044)	(0.020)	(0.031)	(0.023)	(0.032)
adhd	0.190	-0.052	0.006	-0.120	-0.078	-0.323*	0.137	-0.022
	(0.148)	(0.102)	(0.075)	(0.125)	(0.087)	(0.113)	(0.189)	(0.198)
other mental disorders	0.325*	0.306*	0.134*	0.130*	0.126*	0.105*	-0.034	-0.025
	(0.068)	(0.087)	(0.026)	(0.045)	(0.022)	(0.030)	(0.025)	(0.034)
alcohol and drug use disorders	0.558	0.191	-0.592*	-0.061	0.150*	0.142	0.207	0.068
	(0.632)	(0.423)	(0.265)	(0.130)	(0.076)	(0.089)	(0.126)	(0.137)
diabetes	0.377	0.355	0.096	-0.053	-0.048	-0.007	-0.078*	-0.029
	(0.358)	(0.381)	(0.066)	(0.113)	(0.035)	(0.031)	(0.026)	(0.024)
chronic kidney disease		-1.364*	0.582*	0.027	0.183	0.430*	0.388*	0.312*
		(0.545)	(0.278)	(0.391)	(0.201)	(0.143)	(0.105)	(0.072)

Table 6. Regression coefficients and standard errors from Poisson pseudo-maximum likelihood models

	0-19 y. (f)	0-19 y. (m)	20-44 y. (f)	20-44 y. (m)	45-64 y. (f)	45-64 y. (m)	65+ y. (f)	65+ y. (m)
skin and subcutaneous diseases	0.043	0.086	0.122*	0.098*	0.139*	0.062*	0.095*	0.082*
	(0.040)	(0.045)	(0.018)	(0.032)	(0.015)	(0.021)	(0.017)	(0.019)
sense organ diseases	0.101*	-0.023	0.236*	0.209*	0.127*	0.153*	0.137*	0.098*
	(0.036)	(0.040)	(0.020)	(0.033)	(0.014)	(0.018)	(0.017)	(0.018)
rheumatoid arthritis	1.619*	1.050*	0.810*	1.231*	0.558*	0.525*	0.271*	0.290*
	(0.308)	(0.404)	(0.091)	(0.110)	(0.057)	(0.065)	(0.053)	(0.068)
osteoarthritis	1.935*	2.075*	1.044*	1.080*	0.696*	0.827*	0.435*	0.356*
	(0.198)	(0.251)	(0.071)	(0.158)	(0.027)	(0.038)	(0.024)	(0.032)
low back pain	2.185*	1.597*	1.466*	1.776*	1.096*	1.215*	0.836*	0.823*
	(0.222)	(0.245)	(0.058)	(0.089)	(0.037)	(0.045)	(0.032)	(0.047)
osteoporosis	0.900*	2.529*	0.684*	0.700*	0.567*	0.475*	0.383*	0.317*
	(0.384)	(0.423)	(0.085)	(0.179)	(0.027)	(0.061)	(0.020)	(0.035)
other musculoskeletal disorders	1.366*	1.291*	0.882*	1.207*	0.778*	0.832*	0.483*	0.411*
	(0.036)	(0.038)	(0.018)	(0.028)	(0.016)	(0.019)	(0.019)	(0.019)
oral disorders	0.230*	0.441*	0.093*	0.118*	0.144*	0.033	0.130*	0.122*
	(0.070)	(0.094)	(0.040)	(0.060)	(0.035)	(0.050)	(0.035)	(0.048)
other non-communicable diseases	0.518*	0.844*	0.313*	0.409*	0.182*	0.281*	0.235*	0.267*
	(0.098)	(0.179)	(0.024)	(0.061)	(0.016)	(0.021)	(0.016)	(0.019)
well care	0.185*	-0.218*	0.004	-0.950*	0.082*	-0.709*	0.232*	-0.354*
	(0.037)	(0.048)	(0.016)	(0.063)	(0.013)	(0.063)	(0.017)	(0.072)
Constant	3.027*	3.033*	3.951*	3.507*	4.440*	4.214*	4.528*	4.805*
	(0.020)	(0.024)	(0.013)	(0.017)	(0.012)	(0.013)	(0.019)	(0.018)
Adjusted R^2	0.08	0.07	0.16	0.12	0.19	0.19	0.18	0.17
Observations	66567	71059	127532	120801	106368	102632	61884	52945

b coefficients; se in parentheses; f: female, m:male

* $p < 0.05$

The adj. R2 was defined as 1-[(1-R2)*(n-1)/dof]; R2 was defined as the correlation between observed and predicted values on the original scale

7 Regression output: Other outpatient

Table 7. Regression coefficients and standard errors from Poisson pseudo-maximum likelihood models

	0-19 y. (f)	0-19 y. (m)	20-44 y. (f)	20-44 y. (m)	45-64 y. (f)	45-64 y. (m)	65+ y. (f)	65+ y. (m)
HIV/AIDS	-0.865	0.361	0.365	0.098	1.073*	1.049*	1.996*	0.804*
	(0.492)	(0.552)	(0.910)	(0.635)	(0.390)	(0.240)	(0.357)	(0.338)
other communicable diseases	0.271*	0.255*	0.263*	0.289*	0.424*	0.542*	0.338*	0.493*
	(0.048)	(0.036)	(0.053)	(0.103)	(0.058)	(0.098)	(0.047)	(0.057)
hepatitis			1.964*	1.526*	1.744	1.183*	-0.108	1.310*
			(0.871)	(0.509)	(0.905)	(0.334)	(0.389)	(0.615)
maternal and neonatal disorders	1.000*	1.108*	0.289		-0.186			
	(0.164)	(0.117)	(0.260)		(0.576)			
nutritional deficiencies	0.084	0.238*	0.161*	0.031	0.184*	0.605*	0.556*	0.822*
	(0.091)	(0.096)	(0.068)	(0.356)	(0.091)	(0.160)	(0.078)	(0.110)
colon and rectum cancers	-0.848*		-0.319	-2.095	-0.250	0.562*	0.366*	0.213
	(0.217)		(0.543)	(1.364)	(0.291)	(0.251)	(0.145)	(0.193)
trachea, bronchus, and lung cancers	0.397	-1.010	0.234	1.760	0.248	0.832*	0.408	0.015
	(0.230)	(0.785)	(0.498)	(1.427)	(0.308)	(0.252)	(0.228)	(0.216)
breast cancer			0.751*		0.711*		0.450*	
			(0.306)		(0.104)		(0.095)	
prostate cancer		0.617		0.698		-0.121		-0.111
		(0.336)		(0.783)		(0.331)		(0.122)
other neoplasms	0.536	0.419	0.473	1.768*	1.055*	1.277*	0.282*	0.008
	(0.330)	(0.590)	(0.543)	(0.337)	(0.230)	(0.196)	(0.131)	(0.130)
ischemic heart disease	0.067	-0.020	0.160	0.231	0.704*	0.855*	0.431*	0.449*
	(0.225)	(0.205)	(0.230)	(0.250)	(0.159)	(0.132)	(0.084)	(0.093)
stroke	1.723*	-1.085*	2.946*	0.812	1.105*	-0.663	0.297*	0.135
	(0.024)	(0.021)	(0.730)	(0.655)	(0.397)	(0.530)	(0.114)	(0.144)
hypertensive heart disease			-0.149	1.198	0.931	1.095*	0.849*	-0.818*
			(1.282)	(0.623)	(0.565)	(0.340)	(0.418)	(0.301)
atrial fibrillation and flutter	0.063	0.096	-0.469*	0.870	1.261*	1.282*	0.464*	0.196
	(0.311)	(0.592)	(0.229)	(0.724)	(0.414)	(0.437)	(0.167)	(0.121)
other cardiovascular and circulatory diseases	0.052	0.243	0.306*	0.348	0.375*	0.562*	0.176*	0.275*
	(0.168)	(0.268)	(0.111)	(0.180)	(0.058)	(0.089)	(0.043)	(0.064)

Table 7. Regression coefficients and standard errors from Poisson pseudo-maximum likelihood models

	0-19 y. (f)	0-19 y. (m)	20-44 y. (f)	20-44 y. (m)	45-64 y. (f)	45-64 y. (m)	65+ y. (f)	65+ y. (m)
copd	0.216	1.731*	-0.519	0.679	0.493*	0.697*	0.423*	0.526*
	(0.198)	(0.782)	(0.381)	(0.469)	(0.150)	(0.195)	(0.086)	(0.111)
asthma	0.265*	0.467*	0.120	-0.168	-0.124	0.192	0.104	-0.106
	(0.070)	(0.072)	(0.186)	(0.189)	(0.113)	(0.181)	(0.063)	(0.107)
other chronic respiratory diseases	-0.110	-0.166	0.526*	0.275	0.281	0.248	0.457*	0.469*
	(0.143)	(0.257)	(0.179)	(0.266)	(0.168)	(0.162)	(0.091)	(0.104)
cirrhosis and other chronic liver diseases			2.285	-0.214	0.605	1.495*	0.242	0.170
			(1.226)	(0.521)	(0.350)	(0.236)	(0.225)	(0.215)
other digestive diseases	0.333*	0.344*	0.167*	-0.128	0.130*	0.118	0.006	-0.042
	(0.047)	(0.048)	(0.084)	(0.184)	(0.060)	(0.088)	(0.054)	(0.068)
alzheimer's disease and other dementias	-0.358	-1.122	0.305	-0.287	0.066	0.133	0.153*	0.256*
	(0.371)	(0.657)	(0.257)	(0.257)	(0.113)	(0.113)	(0.063)	(0.076)
parkinson's disease	-1.524	-0.707	1.084*	0.762*	0.212	0.373*	0.371*	0.475*
	(0.801)	(0.943)	(0.453)	(0.376)	(0.190)	(0.166)	(0.100)	(0.120)
epilepsy	0.892*	1.120*	1.147*	0.891*	0.773*	0.171	0.268*	0.504*
	(0.115)	(0.252)	(0.216)	(0.248)	(0.137)	(0.207)	(0.080)	(0.092)
multiple sclerosis	0.375	-12.670*	1.037*	-0.272	0.521*	1.147*	0.032	1.117*
	(0.921)	(0.102)	(0.352)	(0.746)	(0.185)	(0.304)	(0.314)	(0.394)
other neurological diseases	0.440*	0.325*	0.562*	0.488*	0.326*	0.271*	0.146*	0.312*
	(0.137)	(0.096)	(0.081)	(0.134)	(0.088)	(0.106)	(0.064)	(0.088)
schizophrenia	1.338*	3.171*	0.274	0.074	0.652*	0.160	0.528*	0.405*
	(0.450)	(0.666)	(0.278)	(0.300)	(0.187)	(0.199)	(0.148)	(0.168)
depression	1.191*	1.420*	0.778*	1.011*	0.679*	0.657*	0.325*	0.459*
	(0.217)	(0.301)	(0.088)	(0.140)	(0.070)	(0.086)	(0.058)	(0.085)
adhd	0.805*	0.437*	0.549*	0.570*	0.338	0.168	0.333	-0.212
	(0.266)	(0.182)	(0.163)	(0.166)	(0.203)	(0.249)	(0.417)	(0.395)
other mental disorders	0.875*	0.705*	0.623*	0.919*	0.594*	0.625*	0.464*	0.381*
	(0.120)	(0.117)	(0.092)	(0.112)	(0.099)	(0.128)	(0.065)	(0.085)
alcohol and drug use disorders	1.754*	1.706*	1.049*	1.454*	0.624*	0.159	0.453	0.496
	(0.476)	(0.581)	(0.302)	(0.180)	(0.174)	(0.274)	(0.298)	(0.359)
diabetes	3.063*	3.425*	1.878*	2.595*	0.891*	0.362*	0.413*	0.358*
	(0.113)	(0.095)	(0.134)	(0.384)	(0.081)	(0.098)	(0.060)	(0.066)
chronic kidney disease		-1.047*	3.205*	4.334*	2.751*	3.261*	2.792*	2.799*
		(0.420)	(0.676)	(0.631)	(0.250)	(0.212)	(0.148)	(0.120)

Table 7. Regression coefficients and standard errors from Poisson pseudo-maximum likelihood models

	0-19 y. (f)	0-19 y. (m)	20-44 y. (f)	20-44 y. (m)	45-64 y. (f)	45-64 y. (m)	65+ y. (f)	65+ y. (m)
skin and subcutaneous diseases	0.135*	0.214*	0.137*	0.417*	0.083	-0.104	0.100*	0.043
	(0.050)	(0.046)	(0.065)	(0.173)	(0.058)	(0.083)	(0.045)	(0.057)
sense organ diseases	0.390*	0.331*	0.263*	0.197	0.222*	0.158*	0.110*	0.063
	(0.039)	(0.037)	(0.060)	(0.134)	(0.055)	(0.072)	(0.048)	(0.055)
rheumatoid arthritis	-0.603*	-0.058	1.426*	0.534	0.472*	0.011	0.133	0.288
	(0.220)	(0.418)	(0.436)	(0.644)	(0.184)	(0.286)	(0.169)	(0.204)
osteoarthritis	0.040	0.160	-0.161	-0.056	0.014	-0.027	-0.006	-0.272*
	(0.164)	(0.233)	(0.206)	(0.403)	(0.105)	(0.136)	(0.062)	(0.068)
low back pain	0.421*	-0.029	0.390*	1.928*	0.140	0.284*	-0.007	-0.217
	(0.180)	(0.146)	(0.155)	(0.923)	(0.123)	(0.139)	(0.083)	(0.167)
osteoporosis	0.684	2.049*	0.364	0.324	0.162	-0.297	0.032	0.061
	(0.453)	(0.696)	(0.263)	(0.387)	(0.103)	(0.218)	(0.049)	(0.117)
other musculoskeletal disorders	0.200*	0.234*	0.457*	0.388*	0.267*	0.235*	0.130*	0.141*
	(0.049)	(0.057)	(0.045)	(0.091)	(0.066)	(0.077)	(0.053)	(0.056)
oral disorders	0.427*	0.436*	0.337*	0.587*	0.269*	0.136	0.149	0.220
	(0.052)	(0.060)	(0.143)	(0.248)	(0.119)	(0.195)	(0.117)	(0.145)
other non-communicable diseases	-0.298	0.094	0.033	-0.300	-0.022	0.356*	-0.001	0.056
	(0.154)	(0.108)	(0.124)	(0.299)	(0.060)	(0.094)	(0.049)	(0.057)
well care	-0.073	-0.043	-0.093	-0.528*	-0.104*	-0.646*	-0.230*	-0.533*
	(0.049)	(0.030)	(0.060)	(0.114)	(0.046)	(0.093)	(0.048)	(0.108)
Constant	4.265*	4.208*	4.070*	3.954*	4.375*	4.465*	5.153*	5.224*
	(0.024)	(0.021)	(0.035)	(0.095)	(0.038)	(0.048)	(0.053)	(0.057)
Adjusted R^2	0.06	0.12	0.19	0.04	0.11	0.16	0.16	0.21
Observations	66567	71059	127532	120801	106368	102632	61884	52945

b coefficients; se in parentheses; f: female, m:male

* $p < 0.05$

The adj. R2 was defined as 1-[(1-R2)*(n-1)/dof]; R2 was defined as the correlation between observed and predicted values on the original scale
